# Functional hub disruption emphasizes consciousness recovery in severe traumatic brain injury

**DOI:** 10.1093/braincomms/fcad319

**Published:** 2023-11-22

**Authors:** Lydia Oujamaa, Chantal Delon-Martin, Chloé Jaroszynski, Maite Termenon, Stein Silva, Jean-François Payen, Sophie Achard

**Affiliations:** University Grenoble Alpes, Inserm, U1216, Grenoble Institut Neurosciences, 38000 Grenoble, France; University Grenoble Alpes, Inserm, U1216, Grenoble Institut Neurosciences, 38000 Grenoble, France; University Grenoble Alpes, Inserm, U1216, Grenoble Institut Neurosciences, 38000 Grenoble, France; Faculty of Engineering, Biomedical Engineering Department, Mondragon Unibertsitatea (MU-ENG), 20500 Mondragon, Spain; Toulouse NeuroImaging Center, Toulouse III Paul Sabatier University, Inserm, 31062 Toulouse, France; Critical Care Unit, University Teaching Hospital of Purpan, 31059 Toulouse, France; University Grenoble Alpes, Inserm U1216, Grenoble Institut Neurosciences, CHU Grenoble Alpes, 38000 Grenoble, France; University Grenoble Alpes, CNRS, Inria, Grenoble INP, LJK, 38000 Grenoble, France

**Keywords:** graph theory, hub disruption index, disorder of consciousness, severe traumatic brain injury, clinical recovery

## Abstract

Severe traumatic brain injury can lead to transient or even chronic disorder of consciousness. To increase diagnosis and prognosis accuracy of disorder of consciousness, functional neuroimaging is recommended 1 month post-injury. Here, we investigated brain networks remodelling on longitudinal data between 1 and 3 months post severe traumatic brain injury related to change of consciousness. Thirty-four severe traumatic brain-injured patients were included in a cross-sectional and longitudinal clinical study, and their MRI data were compared to those of 20 healthy subjects. Long duration resting-state functional MRI were acquired in minimally conscious and conscious patients at two time points after their brain injury. The first time corresponds to the exit from intensive care unit and the second one to the discharge from post-intensive care rehabilitation ward. Brain networks data were extracted using graph analysis and metrics at each node quantifying local (clustering) and global (degree) connectivity characteristics. Comparison with brain networks of healthy subjects revealed patterns of hyper- and hypo-connectivity that characterize brain networks reorganization through the hub disruption index, a value quantifying the functional disruption in each individual severe traumatic brain injury graph. At discharge from intensive care unit, 24 patients’ graphs (9 minimally conscious and 15 conscious) were fully analysed and demonstrated significant network disruption. Clustering and degree nodal metrics, respectively, related to segregation and integration properties of the network, were relevant to distinguish minimally conscious and conscious groups. At discharge from post-intensive care rehabilitation unit, 15 patients’ graphs (2 minimally conscious, 13 conscious) were fully analysed. The conscious group still presented a significant difference with healthy subjects. Using mixed effects models, we showed that consciousness state, rather than time, explained the hub disruption index differences between minimally conscious and conscious groups. While severe traumatic brain-injured patients recovered full consciousness, regional functional connectivity evolved towards a healthy pattern. More specifically, the restoration of a healthy brain functional segregation could be necessary for consciousness recovery after severe traumatic brain injury. For the first time, extracting the hub disruption index directly from each patient’s graph, we were able to track the clinical alteration and subsequent recovery of consciousness during the first 3 months following a severe traumatic brain injury.

## Teaser

Oujamaa *et al*. followed brain network changes of severe subacute traumatic brain-injured patients during clinical recovery. They used the hub disruption index to quantify the global brain network reorganization. The hub disruption index differentiates fully and minimally conscious patients, underlining the clinical relevance of this index to capture consciousness recovery.

## Graphical Abstract

**Graphical Abstract:**
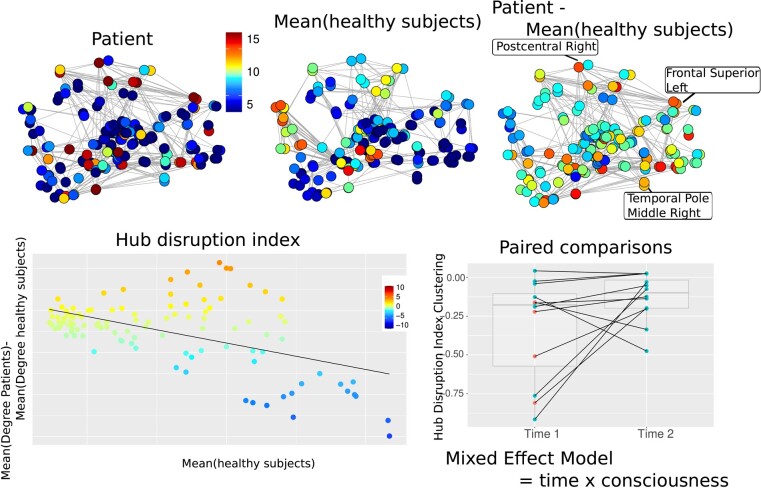

See Edlow and Massimini (https://doi.org/10.1093/braincomms/fcad328) for a scientific commentary on this article.

## Introduction

Severe traumatic brain injury (sTBI) is a worldwide leading cause of mortality and the third cause of acquired neurological disability in adults.^1,2^ Coma occurs secondary to intracranial lesions that compress or directly impair the reticular ascending arousal system (oedema, contusion, haemorrhage) or secondary to traumatic or diffuse axonal injuries (DAIs).^3–5^ The acute sTBI (about 3 weeks) is followed by a subacute (up to 6 months) and a chronic phase (>6 months).^1,6,7^ Arousal reappears first^8^ either associated with recovery of consciousness or not; in the latter case, the patient is subject to transitory or even permanent disorder of consciousness (DOC).^9^ DOC ranges from unresponsive wakefulness syndrome [UWS, previously named vegetative state (VS)] to minimally conscious state (MCS).^10,11^ Emergence from DOC, or consciousness recovery, is considered when the patient recovers either a functional communication or the ability to use two objects from usual life correctly. The poor sensitivity of clinical assessment of consciousness leads to a high rate of misdiagnosis, estimated at 43%.^12^ In other words, clinical tools might fail to distinguish UWS/VS from MCS and MCS from conscious brain-injured patients and to distinguish conscious but fully paralysed patients (namely cognitive motor dissociation syndrome) from UWS/VS patients in 15% of cases in intensive care unit (ICU).^13^ Consequently, the prognosis might be underestimated, leading to limitation of acute care, delayed or denied access to rehabilitation.^14^ To overcome these two issues, functional neuroimaging might complement behavioural assessment by providing a direct measurement of brain activity. Indeed, the scientific communities recommend completing clinical assessment with functional neuroimaging as soon as 28 days post-injury in case of persistent DOC.^9,15,16^

Among the different neuroimaging methods, resting state functional MRI (rs-fMRI) is relevant to investigate the brain networks of patients suffering DOC as it does not require collaboration from non-communicative patients. Comparing all types of DOC (coma, UWS/VS, MCS) to healthy subjects (HS), a reduction of the default mode network (DMN) activity is observed.^17^ Moreover, the level of DMN intrinsic connectivity is reduced and correlate with the severity of DOC.^18,19^ But first the DMN intrinsic connectivity measurement is insufficient to classify DOC patients (UWS versus MCS) and second it is not solely impaired in DOC; other resting state networks’ intrinsic connectivity reduction is reported, specifically in the auditory network, contributing to differentiate MCS and UWS/VS.^20^ So full resting state brain network analysis should be considered in DOC, especially in a longitudinal approach during clinical neurological recovery. Indeed, contrary to persistent DOC, patients recovering consciousness but still severely disabled, might retrieve a physiological extrinsic correlation between DMN and other cognitive resting state networks.^21,22^ Later during clinical recovery, in chronic and fully conscious sTBI patients, intrinsic hyperconnectivity is found in frontal areas of the DMN and in attentional network, stressing the importance of regional analysis of the whole brain network connectivity upon functional networks analysis.^23,24^ Considering TBI pathology, characterized by a high inter-individual heterogeneity in brain lesion distribution, it is important to capture the single subject longitudinal regional changes in connectivity patterns as they might contribute to understand TBI neurological recovery. Yet, resting state studies on DOC are difficult to conduct and are not sufficient to determine whether brain networks derived from rs-fMRI analysis could diagnose or predict consciousness recovery.^25,26^ In this context where longitudinal data are crucial, few studies are currently available sub-acute TBI suffering DOC.^27,28^ Using different methodologies, two studies report a trend towards restoration of a physiological whole brain connectivity pattern while patients recover consciousness.^27,28^

A whole brain network analysis is affordable with graph theory, a mathematical tool fitting to explore resting state networks while taking in account the global topology of the brain connectivity.^29^ Human brain network presents a small world property^30^: a combination of a majority of locally connected nodes (non-hubs) and few nodes linked by sparse long-range edges (hubs). Interestingly after TBI, the brain network loses some integration and enhances its segregation properties.^31,32^ To capture such topological changes after TBI, regional (or nodal) graph metrics [clustering, betweenness centrality (BC), degree] would be more accurate and more reliable than global ones (modularity, small worldness).^33^ Only one study reported longitudinal data acquired between first days in ICU and 6 months after sTBI using graph analysis.^28^ This study supports the view of a close link between normalization of clustering properties in some DMN nodes and recovery of consciousness.

Achard *et al.* applied the graph theory to post-anoxic UWS/VS patients rs-fMRI data, revealing a deep topological disruption.^34^ More precisely, integration and information measures did not differ in average between UWS/VS and HS but at the nodal level a large reorganization of the network was observed: some hubs were lost while some non-hubs became hubs in UWS/VS patients. This nodal level graph modification seems to be particularly relevant in TBI as this pathology might induce a pattern of simultaneous hyper and hypoconnectivity in the global brain network.^24^ Therefore, to quantify such hubs disruptions in the graph, a specific index has been proposed, the hub disruption index (HDI)^34^; which reliability has been confirmed in brain-injured patients.^35,36^ The HDI is pertinent to characterize the functional reorganization of acute anoxic DOC patients as shown by Achard *et al.*^34^ and replicated by Malagurski *et al.*^36^

The present study relies on the hypothesis that functional brain network topology is severely disrupted in DOC subacute sTBI patients, reflected by abnormal segregation and integration nodal values as resumed with the HDI and that along consciousness recovery the network topology returns to normal, reflected by HDI change. Taking advantage of cross-sectional and longitudinal clinical data as well as mathematical theoretical development, the objective of the present study was to answer two main questions: (i) Which connectivity features (integration, segregation and centrality regional values resumed with the corresponding HDI measures) differentiate subacute sTBI patients who recovered consciousness from those still in DOC when discharged from ICU to post-intensive care rehabilitation ward (PICR)? (ii) Do these connectivity features change as the patient recovers consciousness during rehabilitation?

## Materials and methods

### Participants

Patients were recruited in ICU at Grenoble Alpes University Hospital. When inclusion criteria were met and informed written consent obtained from legal surrogates, patients were included in the study. Inclusion criteria comprised age of 18 or older at injury onset, hospitalization in ICU for an acute sTBI (Glasgow Coma Scale score <9 at first medical assessment on the scene)^37^ either isolated or with polytrauma and recovery of a medical condition allowing discharge from ICU (meaning at least 7 days after sedation withdrawal and not requiring artificial life support anymore: no ventilatory support, no haemodynamic support, no dialysis). Patients’ neurological status at inclusion was either conscious or MCS or UWS/VS. Exclusion criteria were medical condition incompatible with MRI, neurological or psychiatric disorders prior to sTBI or no informed consent from patients’ legal representative or sedation continuing. This prospective monocentric study was conducted at Grenoble Alpes University Hospital between February 2015 and March 2018. The protocol was approved by the Ethics committee of the University Hospital of Grenoble Alpes, France (Comité Consultatif pour la Protection des Personnes Sud Est V, ID-RCB 2014-A01873-44/1). The study was registered on ClinicalTrials.gov (Identifier: NCT02647996).

**Figure 1:**
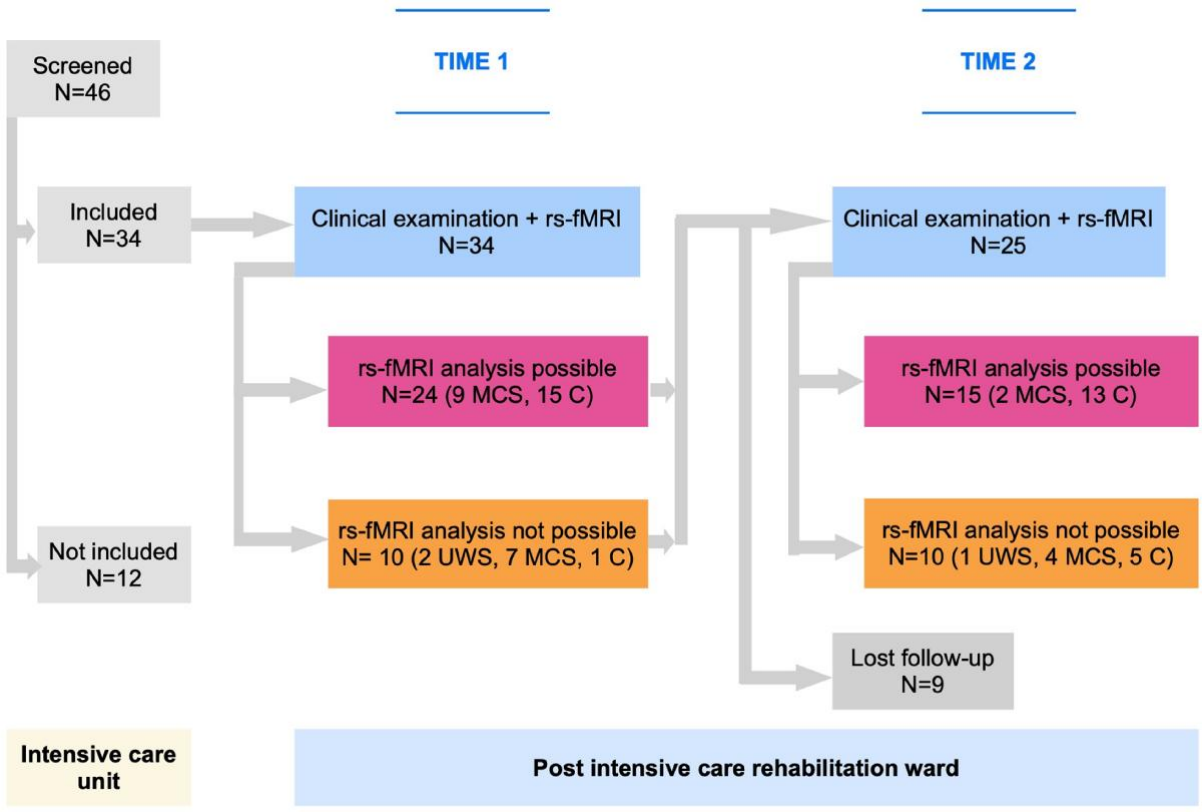
**Flow chart according to time (*x*-axis): 34 sTBI patients were included in this study while staying in ICU.** After admission in PICR (Time 1) and before discharge from PICR (Time 2), clinical evaluations (CRS-R and DRS) and rs-fMRI were acquired. At Time 1, 34 patients were tested providing 24 rs-fMRI datasets with possible analysis and 10 datasets without possible analysis. At Time 2, 25 patients were tested providing 15 rs-fMRI datasets with possible analysis and 10 datasets without possible analysis. rs-fMRI, resting state functional MRI; UWS, unresponsive wakefulness syndrome/vegetative state; MCS, minimally conscious state; C, conscious state.

Among the 46 patients consecutively screened in ICU, 34 corresponded to the criteria and were included in this study (24 males, 10 females, mean age = 38 years old, SD = 16, range 19–67) (Fig. 1). Their initial Glasgow Coma Scale ranged from three to eight (mean = 5, SD = 2) reflecting the severity of the injury. The injury description for each patient was assessed by an expert neuroradiologist not involved in this study following their first MRI scan including T_1_ and eventually FLAIR sequences if available (Table 1). As each patient was comatose immediately and durably after TBI, we assumed they all suffered DAI, with or without additional grey matter (GM) Contusions (DAI ± C). In this study, the rs-fMRI data of 20 age-matched healthy participants (all men, mean age 41 years old, SD 11, *t*-test Student for age: *P* = 0.48), but not gender matched (Fisher's test: *P* = 0.009), were used from another research protocol.^38^ Their data were acquired on the same MRI scanner with the same sequences and at the same period to ensure that the settings of the scanner were identical.

**Table 1:** Demographic and injury characteristics of the patients

Patients	Age	Gender	Lesion description	GCS
1	35	M	DAI (mainly corona radiata and splenium)	3
2	23	M	DAI	4
3	27	M	DAI (mainly R hemisphere)	3
4	54	F	DAI	4
5	55	M	DAI + bilateral fronto-basal and medial parietal contusions + VM	4
6	44	M	DAI + R fronto-lateral contusion + VM	3
7	29	M	DAI (mainly fronto-orbital)	8
8	23	M	DAI (occipital) + L fronto-basal and temporal contusions	7
9	64	M	DAI + R fronto-temporal contusion + diffuse SAH	6
10	22	F	DAI (mainly corpus callosum)	7
11	31	M	DAI + R superior frontal contusion	6
12	20	F	DAI + R frontal contusion + R sinking skin flap syndrome	6
13	40	M	DAI	5
14	54	F	DAI + L frontal and R occipitoparietal contusions + VM	6
15	27	M	DAI	7
16	66	F	DAI + L temporal, medial bifrontal contusions + falx cerebri SAH	8
17	19	M	DAI (mainly vertex) + R parieto-occipital contusion	3
18	43	M	DAI + full ischemic damage on L sylvian territory + L craniectomy	5
19	45	M	DAI + mesencephalic, and R cerebellar contusions, L craniectomy	3
20	54	F	DAI + L frontal and temporal contusions, L craniectomy	8
21	67	M	DAI + R frontal and bilateral temporal contusions, occipital SAH	8
22	20	M	DAI + R temporal contusion, diffuse SAH, R craniectomy	5
23	38	M	DAI (mainly bifrontal)	6
24	33	F	DAI (mainly corpus callosum) + R parietal contusion + L SDH	8
25	19	F	DAI (mainly medial-anterior frontal lobes and corpus callosum)	5
26	24	M	DAI (mainly vertex)	6
27	47	M	DAI + L frontal contusion	8
28	32	M	DAI (mainly frontal bilateral) + L precentral gyrus contusion	3
29	51	F	DAI (+L SDH)	3
30	65	M	DAI (+bilateral frontal SDH)	3
31	51	M	DAI (mainly R frontal) + R frontal contusion + L frontal SDH	4
32	54	F	DAI + diffuse SAH	7
33	20	M	DAI (mainly R frontal and L external capsule)	7
34	23	M	DAI + bilateral frontal contusions	8

DAI, diffuse axonal injury; R, right; L, left; VM, ventriculomegaly; SDH, subdural hematoma; SAH, subarachnoid haemorrhage; GCS, Glasgow coma scale.

### Protocol

The timing for MRI examinations was corresponding to the usual medical pathway of acute sTBI patients in our trauma centre (Fig. 1). Time 1 was the date of admission to PICR immediately following discharge from ICU. Time 2 was the date of discharge from PICR to conventional rehabilitation centre. These timing matched approximately with 1 and 2 months post-sTBI, respectively,^39^ but could be shortened or extended depending on patient’s medical issues and bed availability. In France, the PICR unit is dedicated to severely brain-injured patients who do not require intensive care anymore but are still requiring close medical management to prevent and treat frequent medical complications. These patients are severely disabled with need for technical support like bronchial secretion suction by tracheostomy and gastrostomy feeding.

The experimental protocol included a cross-sectional and a longitudinal study. In the cross-sectional study, at Time 1, the sTBI group was subdivided in two groups according to their consciousness state (MCS or conscious): the MCS-group (as no UWS/VS rs-fMRI acquisition reached the quality level requested; Fig. 1) and the C-group. The brain networks between them were compared. This design was to answer the question of a differential graph topology related to consciousness state.

In the longitudinal study, a group comparison was done between admission to PICR (Time 1) and discharge from PICR (Time 2). During their PICR stay, sTBI patients received a daily multidisciplinary rehabilitation to promote wakefulness, communication and self-care independence.^40,41^ This design was to answer the question of whether a graph topology changes or not during the subacute stage of the sTBI.

At Time 1 and 2, the assessments consisted of clinical measurement of consciousness and neurological disability, as well as an rs-fMRI examination, all done the same day.

### Clinical evaluations

At Time 1 and 2, consciousness was assessed with the coma recovery scale revised (CRS-R).^42^ The CRS-R scores from 0 (coma) to 23 (conscious). This score comes from the addition of 29 items dispatched under six sub-scores (auditory, visual, motor, oromotor/verbal functions plus communication and arousal scales). The CRS-R diagnoses the state of consciousness by categorizing patients either in coma, DOC (UWS/VS, MCS) or in conscious state (exit-MCS) according to their behavioural responsiveness and communication ability. States of consciousness, CRS-R scores and sub-scores were collected at Time 1 and 2 for each patient (Table 2). See Supplementary Table 1 for details about neurological disability.

**Table 2:** Clinical evaluations of the patients

Time 1				Time 2		
Patients	Delay (day) sTBI—rs-fMRI	Consciousness state	CRS-R score (sub-scores)	Delay (day) sTBI—rs-fMRI	Consciousness state	CRS-R score (sub-scores)
1	90	UWS/VS	5 (111 101)	193	UWS/VS	8 (221 102)
2	**30**	**MCS**	**13** (**235102)**	62	C	22 (446 323)
3	**27**	**MCS**	**10** (**314101)**	**105**	**C**	**22** (**456223)**
4	**24**	**C**	**20** (**346313)**	**73**	**C**	**23** (**456323)**
5	**71**	**MCS**	**12** (**225102)**	151	MCS	20 (445 313)
6	**47**	**C**	**23** (**456323)**	89	C	23 (456 323)
7	**26**	**C**	**23** (**456323)**	**89**	**C**	**23** (**456323)**
8	**20**	**C**	**23** (**456323)**	**83**	**C**	**23** (**456323)**
9	**37**	**MCS**	**15** (**333213)**	**75**	**C**	**23** (**456323)**
10	27	MCS	17 (445 202)	34	MCS	17 (445 202)
11	**13**	**C**	**23** (**456323)**	N/A	N/A	N/A
12	58	MCS	9 (331 002)	85	MCS	12 (223 203)
13	**19**	**C**	**21** (**445323)**	N/A	N/A	N/A
14	51	MCS	9 (223 101)	**104**	**MCS**	**12** (**333102)**
15	**44**	**C**	**23** (**456323)**	**66**	**C**	**23** (**456323)**
16	32	MCS	9 (223 101)	46	C	20 (456 311)
17	**38**	**C**	**17** (**335321)**	**87**	**C**	**23** (**456323)**
18	**69**	**MCS**	**13** (**335101)**	113	C	19 (346 213)
19	**48**	**C**	**23** (**456323)**	**67**	**C**	**23** (**456323)**
20	**43**	**C**	**23** (**456323)**	N/A	N/A	N/A
21	**23**	**MCS**	**15** (**335211)**	**34**	**C**	**16** (**336211)**
22	61	MCS	8 (113 102)	117	MCS	18 (345 213)
23	**33**	**MCS**	**14** (**335111)**	54	C	23 (456 323)
24	**32**	**C**	**23** (**456323)**	**54**	**C**	**23** (**456323)**
25	**28**	**C**	**23** (**456323)**	N/A	N/A	N/A
26	32	C	17 (435 122)	**54**	**C**	**23** (**456323)**
27	**21**	**C**	**23** (**456323)**	**70**	**C**	**23** (**456323)**
28	**25**	**MCS**	**12** (**325101)**	N/A	N/A	N/A
29	31	MCS	11 (323 102)	**58**	**MCS**	**13**(**333103)**
30	37	UWS/VS	4 (021 100)	N/A	N/A	N/A
31	**47**	**MCS**	**11** (**323201)**	**160**	**C**	**23** (**456323)**
32	33	MCS	15 (335 202)	N/A	N/A	N/A
33	**42**	**C**	**23** (**456323)**	N/A	N/A	N/A
34	**21**	**C**	**23** (**456323)**	N/A	N/A	N/A

Text appears in bold when the rs-fMRI data were fitting for HDI computation.

sTBI, severe traumatic brain injury; rs-fMRI, resting state functional MRI; UWS/VS, unresponsive wakefulness syndrome/vegetative state; MCS, minimally conscious state; C, conscious state; N/A, not applicable; CRS-R, coma recovery scale revised.

### Neuroimaging acquisition and graph construction

The MRI scanning sessions were conducted on a 3 T Philips Achieva-TX scanner (Best, The Netherlands) at the Grenoble MRI facility-IRMaGe, equipped with a 32 channel-head coil.

### MRI acquisitions

After scout images acquisitions, the MRI examination consisted in a structural high resolution T_1_-weighted image (3D MPRAGE) followed by a long resting-state fMRI BOLD-weighted acquisition and an FLAIR, when possible, all recorded without sedation.

The fMRI sequence covered the whole brain. A long acquisition (13′20″ duration) was chosen since it is directly related to the reliability of rs-fMRI connectivity estimates.^35,43^ See Supplementary material for details on MRI parameters.

### Resting state functional MRI pre-processing

Functional data were pre-processed with the SPM12 software for each subject and each timepoint. First, functional images were realigned and corrected for time shift between slices. To detect artefacted images related to head motions, we further used the ART toolbox that detects volumes presenting inter-volume composite motion larger than 5 mm or inter-volume signal change higher than 3SD of the mean signal time course. When an image was artefacted a corresponding outlier was created for further regression. Datasets for which there were more than 10% of outliers were rejected of the analysis. Structural images were then co-registered to the mean functional image and segmented to obtain the GM probability map. This co-registration was checked visually to ensure the quality of this step (Supplementary Figs 1and 2). The GM probability image was normalized elastically using the algorithm DARTEL^44^ to fit individual GM image onto the ICBM152 template. This elastic normalization is of particular interest for patients presenting ventriculomegaly, subdural hematoma or subarachnoid haemorrhage since this algorithm is efficient at correcting the strong dilation of the ventricles or deformations related to hematoma or haemorrhage (which was visually checked here). This procedure computes an elastic deformation field for each participant that was further inversed. Its application to a chosen atlas customizes accurately the atlas onto the individual GM image. Subsequent analysis of brain network was performed in the referential of each participant. The parcels of the customized atlas were eventually applied to the individual fMRI images.

### Parcellation

The atlas we chose for parcellation is the version three of the classical Anatomic-Automatic-Labelling (AAL3) composed of 166 regions.^45^ The AAL3 was chosen as it contains cortical, cerebellar and sub-cortical nuclei. This precision is relevant here since sub-cortical nuclei are implicated in the arousal network. However, one limit of AAL3 is the large heterogeneity of the region sizes ranging from 48 mm^3^ to 41 cm^3^. This led us to pool nuclei within the thalamus to achieve three sub-regions: the medial part, the lateral part and the pulvinar. We pooled the cerebellum in three subparts: the anterior lobe, the posterior lobe and the vermis. The inferior cerebellar lobe was not included since data from these regions were not acquired. Finally, the smallest regions (locus coeruleus, raphe nucleus, red nucleus and substantia nigra) were discarded from the analysis, leading to a final atlas containing 107 regions (Supplementary Table 2).

In each parcel of the customized atlas, mean time series were estimated at each time point by averaging the EPI time series of all voxels of the parcel weighted by the GM probability of these voxels. This weighting limits the contamination of the time series by white matter signals and cerebrospinal fluids. Residual head motion was eventually removed by regressing out motion parameters and outliers previously detected. The motion parameters were computed by group and tested between groups (Supplementary Fig. 3).

### Wavelets decomposition

Resulting time series were decomposed in scales (each corresponding to a different frequency range) using discrete dyadic wavelet transformation.^30^ We applied the maximal overlap discrete wavelet transform to each regional mean time series and estimated the pairwise inter-regional correlations at each of the four wavelet scales. The relevant information for rs-fMRI data, below 0.1 Hz, is mainly contained in the frequency interval 0.032–0.065 Hz.^30^

### Graph computation

All correlations between pairs of regional time series in this frequency interval are further pooled into a correlation matrix for each subject at each time. The mean correlation values were computed per subject and per group and confirmed no differences in correlation values between groups (mean correlation HS = 0.35 [0.25; 0.5], mean correlation sTBI group Time 1 = 0.32 [0.22; 0.48], mean correlation sTBI group Time 2 = 0.30 [0.19; 0.5]; Kruskal–Wallis test: *P* = 0.08).

To compute the graph of brain network, we first extracted the minimum spanning tree based on the correlation matrix to keep the graph fully connected.^46^ The remaining absolute values of correlation matrices were thresholded to create an adjacency matrix that defines an unweighted and undirected graph for each subject at each time. We choose to preserve the power of the study (to keep all the patients in the study) by reducing the cost of the graph at 5% corresponding to 283 edges.

### Graph metrics computation

Each metric gives a particular description of the topology of the graph (Supplementary Table 3). They can be computed at different levels: giving information at the global level (global metrics), about clusters inside the graph (intermediate metrics) or about each node (nodal metrics). Since the reorganization of the brain network addressed may contain both disconnections and over-connections, we investigated the nodal metrics with a focus on how the properties of the graphs were changed in relation with sTBI. For each node, several metrics can be extracted representing different characteristics of nodal connectivity.^33^ The clustering (or local efficiency) metric relies on the connectivity property in the direct neighbourhood of a node thus corresponding to a segregation property. The degree metric, the number of connections that link a node to the rest of the network thus corresponding to an integration property. The BC of a node represents how many of the shortest paths between all other nodes pass through this given node. A hub is defined as a node occupying a central position in the overall organization of a network.^47^ Relatively to the metrics we chose, a hub is defined as a node with high clustering and/or high degree and/or high betweenness value. To extract these metrics, we used brainwaver and igraph R libraries, tools that are freely available on CRAN (https://cran.r-project.org/web/packages/brainwaver).

### Hub disruption index computation

**Figure 2:**
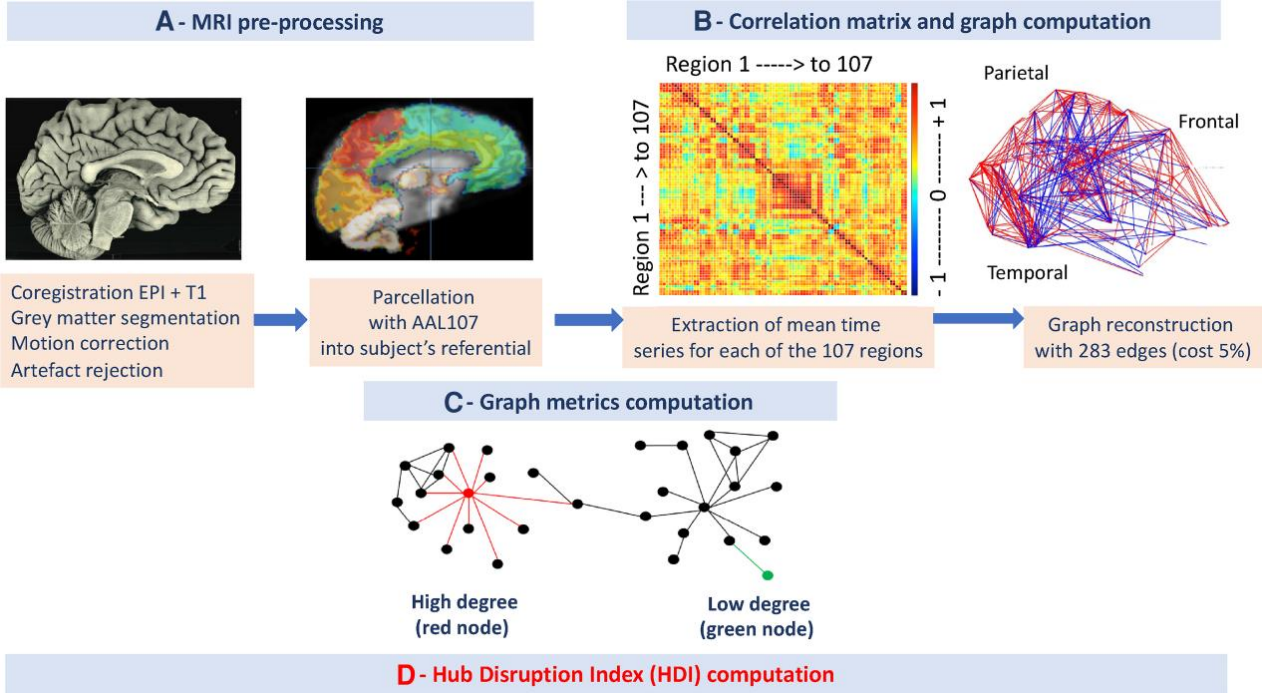
**Methodological steps from MRI pre-processing (A) to HDI computation (D).** MRI pre-processing steps from T_1_ and echoplanar imaging (EPI) sequences co-registration to parcellation shown in **A**. Correlation matrix computation is done using mean time series of each region to extract the graph composed of 107 nodes and 283 edges (or connections) between nodes when thresholded at 5% cost shown in **B**. Illustration of the metric degree with a network comprising a node of high degree value (left: 11 edges) and a node of low degree (right: one edge) shown in **C**. HDI computation shown in **D** (see Fig. 4).

**Figure 3:**
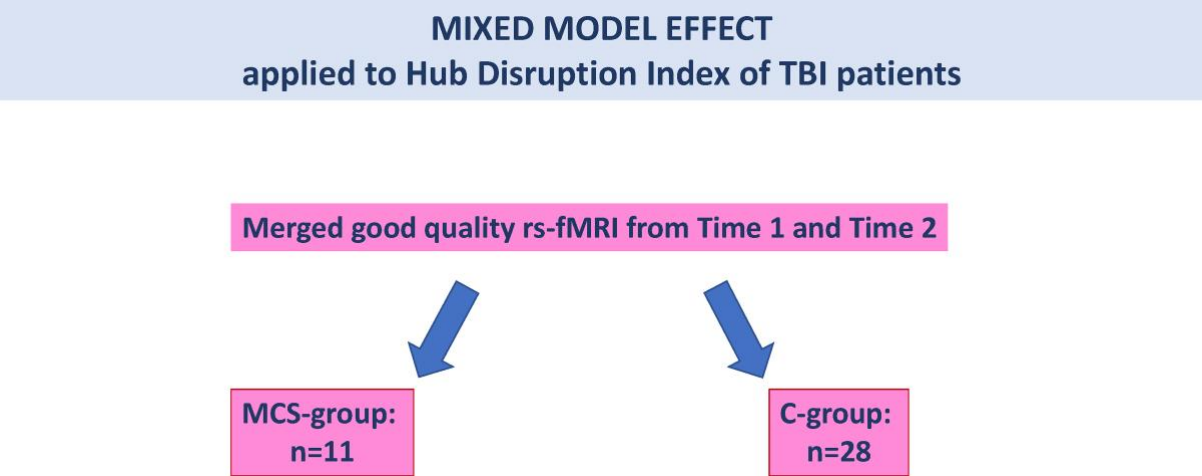
**Statistical mixed model effect applied to HDI results.** To pursue sTBI rs-fMRI data analysis with the mixed model effect, we pooled all timepoints and shared data between minimally conscious (MCS) and conscious (C) groups.

**Figure 4:**
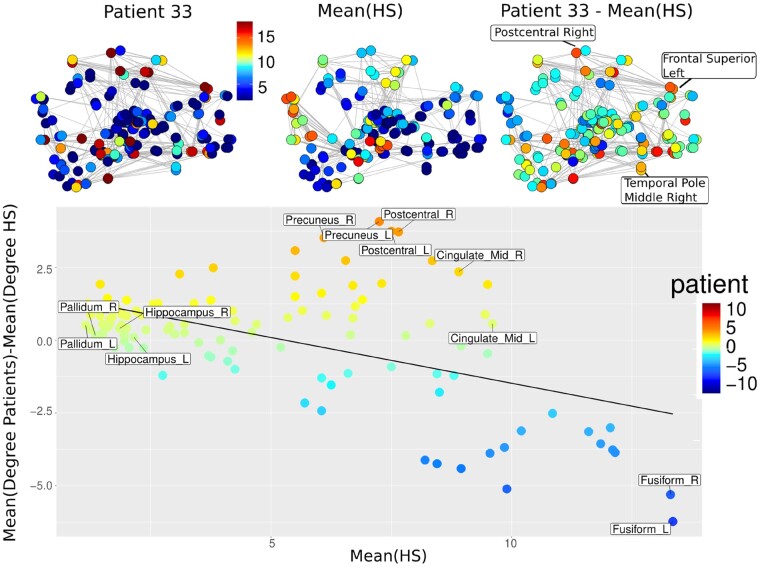
**HDI computation.** Example of sTBI patient 33 for the metric degree. On *upper row* the HDI computation: the graph of this subject is represented with coloured circles corresponding to the value of the metric degree for each node (left graph). Then it is compared to the mean graph of a group of HS (*middle*) providing a differential subject versus HS’ graph (*right*). Then (*lower row*) for each node, this differential degree value for patient 33 is reported in *y*-axis, while its mean degree value for HS is reported in *x*-axis. The slope of the regression line of this plot represents the HDI-degree of patient 33. In case of an individual’s graph not different from the mean graph (e.g. an HS), nodal differences values are close to 0 and the regression line between all these nodes is horizontal with a slope about zero (HDI = 0). When an individual’s graph is different from the mean graph, as in this example patient 33, the regression line between the nodes is decreasing with negative slope (HDI < 0), meaning that hubs in the patient’s graph (in the *right part* of the plot) have reduced values while non-hubs in the patient’s graph (in the *left part* of the plot) present increased values.

The HDI^34^ quantifies the comparison of the distribution of the nodal metrics in an individual graph with respect to the distribution of the nodal metrics in a referential graph (corresponding to the mean graph of a group of HS). The HDI were computed for all nodal metrics, providing an HDI-clustering, HDI-degree and HDI-BC. Its computation was performed for each individual graph (Figs 2–4). In case of an sTBI patient, the graph may be modified due to the injury and nodes that behave as hubs in the referential graph (with high degrees) present reduced degree values, while other nodes that are not hubs (with low degrees) present increased degree values. The pattern of increased nodal metrics in non-hubs and decreased nodal metrics in hubs is resumed in the HDI. The higher the HDI (in absolute value), the larger the difference with the reference. The HDI thus resumes the overall nodal differences with a referential graph.

To guess what HDI corresponds to, we suggest an analogy with the airline network. If a hub airport becomes dysfunctional (loses its connectivity with other airports), then other smaller airports usually connected with the hub will increase their connections (or connectivity) with other airports to keep the whole network properties. Consequently, there will be both hypoconnectivity in an airport and hyperconnectivity in others. This double pattern of hypo- and hyper-connectivity is summarized by the HDI. The more hubs are dysfunctional, the highest the reorganizations. Thus, HDI is a global indicator of the whole network reorganization.

### Statistical analysis

At admission in PICR (Time 1), to test the main hypothesis, that is a different value of the HDI between C-group and MCS-group, a non-parametric Mann–Whitney test was used. To further compare the HDI in these patient groups with HS, a Kruskal–Wallis test was performed. The potential influence of the lesions on the HDI was eventually addressed according to the lesion type (DAI or DAI + C) with a Mann–Whitney test. The mean metric values (clustering, degree, BC) between HS and each group of patients were tested using Kruskal–Wallis test.

Participants’ trajectories were modelled using linear mixed-effects model including state of consciousness, time and their interaction (fixed effects), and subject-specific random effects. The predictors of interest were state of consciousness and its interaction with time to investigate whether HDI changes differed between states of consciousness. A linear mixed-effect model fit by REML (restricted maximum likelihood)^48^ was applied to quantify intra and inter subjects’ HDI change. We provide confidence intervals and *P*-values using bootstrap techniques as implemented in the package parameters.

To explore the regions accounting for mean HDI difference between HS and C-group, HS and MCS-group and finally MCS-group and C-group, we performed Wilcoxon rank tests.

Statistical analyses were run with the support of Grenoble University Hospital statistical team.

## Results

### Network disruption in severe traumatic brain injury patients at discharge from intensive care unit

The cross-sectional study took place at Time 1, corresponding to discharge from ICU to PICR (Table 2). At this time, the rs-fMRI data of 24 patients could be fully analysed while those from 10 patients were excluded for excessive head motion or strong distortion of skull and brain compromising co-registration (Fig. 1). The state of consciousness was more altered in the group of patients whose data were excluded (Fisher’s test, *P* = 0.006) (Fig. 1). The demographic and clinical characteristics of these 24 patients were 20/4 ratio men/women; mean age 38 ± 14 years; mean initial GCS score 6 ± 2; delay between sTBI and first MRI examination 35 ± 15 days (13–71); mean CRS-R total scores 19 ± 5. None were UWS/VS, nine were in MCS (MCS-group) and 15 conscious (C-group) according to the CRS-R assessment at Time 1 (Tables 1 and 2).

**Figure 5:**
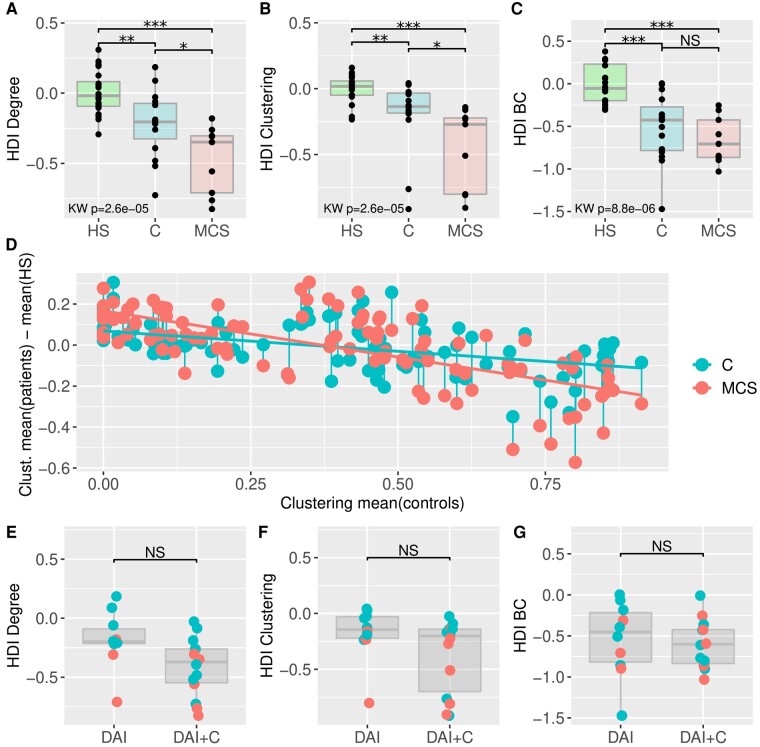
**HDI at Time 1.** HDI at discharge from the ICU (Time 1). The patients that are in minimal conscious state (MCS) present a significantly stronger brain network reorganization than conscious patients (C) as compared to HS brain networks. This is found both with Degree metric (integration property of the graph) (**A**) and with clustering network (segregation property of the graph) (**B**) but is not significant with BC metric (hubness properties) (**C**). (**D**) For each node, the averaged metric among all conscious patients and among all minimally conscious patients is plotted. The HDI is computed as the slope of the nodal metric among all 107 regions. The slope is steepest for MCS patients as compared to C patients, meaning that brain networks’ reorganization in MCS is larger than in conscious patients. No significant differences in HDI metrics were found according to disease status [diffuse axonal injury (DAI) alone or diffuse axonal injury with contusions (DAI + C)] in **E**–**G**. Statistical tests are Mann–Whitney tests for paired comparison and significance level is consequently indicated by stars: * = 0.05 ** = 0.005 *** = 0.0005 while NS indicates non-significant test. Then a Kruskal–Wallis test is applied for multiple group comparison and the corresponding *P*-value is provided.

At Time 1, the HDI-clustering was relevant to significantly distinguish HS, MCS and C-group (HDI-clustering C-group = −0.14 [−0.19; −0.03], HDI-clustering MCS-group = −0.27 [−0.8; −0.22]; Mann–Whitney test C-group versus MCS-group: *P* = 0.014) (Fig. 5B). The HDI-degree also discriminated HS, MCS and C-groups (HDI-degree C-group = −0.2 [−0.32; −0.07], HDI-degree MCS-group = −0.35 [−0.71; −0.30], Mann–Whitney test C-group versus MCS-group: *P* = 0.022) (Fig. 5A). The HDI-BC was not statistically different between the two groups of patients (HDI-BC C-group = −0.43 [−0.78; −0.27], HDI-BC MCS-group = −0.71 [−0.86; −0.42], Mann–Whitney *P* = 0.26) (Fig. 5C). Altogether, these results indicate that DOC relates to local and global disruption of functional connectivity, interpreted as reconfiguration of segregation and integration properties of the graph.

Opposite to the topological disruption analysis, the means of all nodes’ metrics (clustering, degree, BC) were not differentiating HS from sTBI-group, neither MCS nor C-groups. That may be due to the concomitant increased values in some nodes and decreased values in others, as found in anoxic UWS/VS patients.^34^ To further identify the nodes where clustering was increased or decreased, we compared the plots of clustering values for all nodes, which permitted the computation of HDI-clustering for C-group (in light blue) and MCS-group (in light red) in Fig. 5D. Those regions are provided in Supplementary Table 7.

We also compared patients according to their brain lesions, categorizing them either with pure DAI (*n* = 10) or with DAI plus GM contusion (DAI + C, *n* = 14) (Table 1). These two groups do not differ, whatever the metric considered (HDI-degree, HDI-clustering, HDI-BC) (Fig. 5E–G). We found that the significant group difference in HDI-clustering is not driven by the type of lesions. These important findings allowed us to merge the graph data from the two types of injuries (DAI or DAI + C) in further analyses.

### Longitudinal network disruption in severe traumatic brain injury patients

The longitudinal study is exploring the transition from Time 1 to 2 (from ICU discharge to PICR discharge) (Table 2). At Time 2, the rs-fMRI data of 15 patients were fully analysed while 10 patients were excluded for excessive head motion, strong distortion of skull and brain compromising co-registration (Fig. 1). Considering the proportion of UWS/VS, MCS and C patients according to the CRS-R, the state of consciousness was not different between the group whom data were excluded (*n* = 10) and those analysed (*n* = 15) (Fisher’s test, *P* = 0.09).

These 15 patients (11 men, aged 41 ± 16 years old, 19–67) had an initial Glasgow Coma Scale score of 6 ± 2, a delay between sTBI and rs-fMRI of 80 ± 32 days (34–160) (Tables 1 and 2). None were UWS/VS, two were MCS and 13 conscious according to the CRS-R at Time 2. The mean CRS-R total score was 21 ± 4 (Table 2). From Time 1 to 2, the number of MCS in diminished (Time 1: *n* = 24 comprising nine MCS and 15 C; Time 2: *n* = 15 with two MCS and 13 C). Among the 15 patients’ rs-fMRI data available at Time 2, 12 were paired (for each patient, data from Time 1 and 2 were available). The paired patients (10 men, two women, aged 40 ± 16 years old) had a mean initial GCS 6 ± 2, a mean delay between sTBI and rs-fMRI of 80 ± 31 days. Among these 12 paired patients, four were MCS and eight were conscious at Time 1, and all were conscious at Time 2.

**Figure 6:**
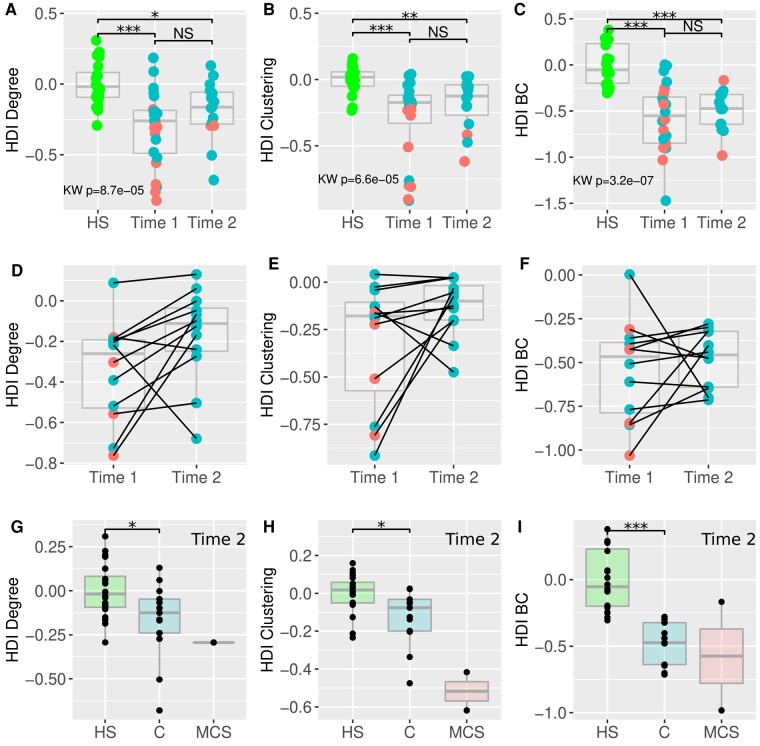
**HDI at Time 2 and its evolution.** *Upper row*: comparison of the HDI between HS, patients at discharge from ICU (Time 1) and patients at discharge from PICR (Time 2) for HDI-degree (**A**), HDI-clustering (**B**) and HDI-BC (**C**). *Middle row*: comparison of the HDI between the 12 paired patients at discharge from ICU (Time 1) and at discharge from PICR (Time 2) for HDI-degree (**D**), HDI-clustering (**E**) and HDI-BC (**F**). Note that for the HDI-degree (**D**) the two values available are very closed so that the plots are overlapping. *Lower row*: comparison of the HDI between HS, C-group and MCS-group at discharge from PICR (Time 2) for HDI-degree (**G**), HDI-clustering (**H**) and HDI-BC (**I**). The MCS sample at Time 2 (*n* = 2) does not allow statistical analysis but is provided here for descriptive purpose. MCS, minimally conscious state; C, conscious state. Statistical tests are Mann–Whitney tests for paired comparison and significance level is consequently indicated by stars: * = 0.05 ** = 0.005 *** = 0.0005 while NS indicates non-significant test. Then a Kruskal–Wallis test is applied for multiple group comparison and the corresponding *P*-value is provided.

From Time 1 to 2, the sTBI group did not change significantly considering HDI for degree, clustering and BC (Fig. 6A–C). Nevertheless, the HDI values for the three metrics tended to evolve towards the HDI value of HS from Time 1 to 2.

The paired Wilcoxon rank test performed with the rs-fMRI data of the 12 patients whose graph could be analysed at both timepoints showed a significant difference for HDI-degree (*P* = 0.04) but not for HDI-clustering (*P* = 0.09), nor for HDI-BC (*P* = 0.23) (Fig. 6G–I).

At Time 2, the C-group still presented a significant difference with the HS (Wilcoxon rank test *P* = 0.008 for clustering, *P* = 0.04 for degree and *P* < 0.0001 for BC) (HDI-degree C-group = −0.17 [−0.68,0.13]; HDI-clustering C-group = −0.12 [−0.47,0.024]; HDI-BC C-group = −0.48 [−0.71, −0.28]) (Fig. 6D–F).

### Modelling network disruption in severe traumatic brain injury

The sensitivity of HDI to consciousness state was explored with the mixed effect model applied to the whole cohort (*n* = 39; MCS = 11, C = 28) (Fig. 3). The state of consciousness explained the topological connectivity changes (HDI evolution for clustering and degree) during follow-up, while time did not (HDI-clustering for consciousness: *P* = 0.034; HDI-clustering for time: *P* = 0.31; HDI-degree for consciousness: *P* = 0.026; HDI-degree for time: *P* = 0.356). Considering one or the other metric, the interaction between consciousness and time was not significant (Table 3).

**Table 3:** Mixed effect model

HDI = effects + effects ∗ Consciousness + effects ∗ Time + effects ∗ Consciousness × Time + randomintercep + randomeffect			
	Coefficient	95% CI	*P*-value
HDI-clustering			
Intercept	−0.20	[−0.33, −0.08]	0.008
Consciousness	−0.24	[−0.44, −0.02]	**0.034**
Time	0.09	[−0.09, 0.26]	0.310
Consciousness and time interaction	−0.16	[−0.61, 0.25]	0.454
HDI-degree			
Intercept	−0.23	[−0.35, −0.12]	<0.001
Consciousness	−0.23	[−0.43, −0.03]	**0.026**
Time	0.08	[−0.07, 0.24]	0.356
Consciousness and time interaction	0.11	[−0.30, 0.51]	0.628

According to the mixed effect model, a conscious sTBI patient has an HDI absolute value (clustering, degree) lower than an MCS sTBI patient (Table 3). Because they do not reach statistical significance, we report the results of the model for HDI-BC and for modularity in Supplementary Table 4.

An exploratory analysis on the regions implicated in graph disruption was conducted. The detailed results are reported in Supplementary Figs 7–9 and Tables 5–7. After correction for multiple comparison analysis, no single region accounted for HDI change between clinical conditions (MCS versus conscious TBI, MCS versus healthy control, conscious TBI versus healthy control). Of note left parahippocampal and several left occipital regions tend to reach significance for HDI-clustering change between MSC and conscious TBI patients.

## Discussion

Using graph analysis applied to rs-fMRI, we quantified the brain network topological disruption and its longitudinal change after sTBI. Doing so we showed how hub disruption emphasizes consciousness recovery. During sTBI subacute stage, a time window of intense brain plasticity,^49^ substantial modifications occurred in patients’ brain network, when compared to HS, leading to patterns of both hyper- and hypo-connectivity.^24^ Here, using the HDI, a metametric that resumes the simultaneous hyper- and hypo-connectivity patterns, we quantified changes in the brain network properties both for transversal and longitudinal analysis. Such data are complex to acquire and consequently rare. The HDI (clustering and degree) significantly discriminated the fully conscious patients from those with a minimal consciousness. In addition, we observed that brain network reorganization was partly reversible with consciousness recovery as assessed by partial reduction of HDI values. This HDI reduction towards mean value of HS was related to consciousness state rather than time as shown by a mixed effect model. Along consciousness recovery, the HDI-clustering partial reduction might be driven by restoration of normal clustering values in DOC lost hubs. As far as we know we are the first to report significant functional hub disruption in a subgroup of sTBI with pure axonal injury (Fig. 5E–G). The DAI might explain a reduced activity in hubs of DOC patients in acute stage after sTBI.^50,51^ DAI also alter functional centrality properties of the posterior cingulate cortex of chronic conscious TBI.^31^

### Subacute severe traumatic brain injury: a crucial period for clinical improvement

In the sTBI subacute stage, the synaptic plasticity necessary for functional recovery will be facilitated or inhibited depending on the intensity of microglial response to blood–brain barrier damage and neuronal death.^52^ This complex astrogliosis reaction can promote or complicate neurogenesis, synaptogenesis and angiogenesis necessary to restore neuronal activity.^53,54^ Along with these modifications at a microscopic level, brain activity evolves at the macroscopic level while clinical recovery occurs.^55^ During this subacute stage, the literature, although sparse, suggests the retrieval of a normal intra DMN functional connectivity pattern with gradual consciousness recovery.^27,56^ Our study took place in this subacute period crucial for clinical improvement like consciousness recovery.^9,15,16^ First, we show a wide change of segregation and integration properties of brain network 1-month post severe TBI. Secondly, we show a concomitant restoration of segregation and integration properties of brain network and consciousness 3 months after sTBI. We were able to show first that these functional connectivity disruptions were largely encompassing the DMN and second that they were related to consciousness recovery. Apart us, only two studies reported longitudinal data acquired between first days and 6 months after sTBI, only one of them using graph analysis.^27,28^ Although employing different methodological approaches, these studies support the view of a close link between normalization of the functional connectivity pattern and recovery of consciousness. Indeed, DMN functional extrinsic hypo-connectivity was restored in Threlkeld’s seed-based approach while clustering properties improved in selected nodes of the DMN in Crone’s work. Of note Crone *et al.* checked the stationary functional connectivity and failed to report any functional connectivity difference according to consciousness level. We did find a difference using stationary functional connectivity in the present work, supporting the interest of the HDI.

### After a severe traumatic brain injury, brain network presents patterns of both hypo- and hyper-connectivity

If DMN hypo-connectivity is consistently reported in DOC,^17,18,20,57–61^ hyper-connectivity is also reported in the anterior part of the DMN,^58^ in the posterior cingulate region,^62^ in the limbic network^21^ and between cognitive resting state networks (DMN and Task positive network).^22,63^ These discrepancies point the methodological limits consisting of merging subacute and chronic traumatic and non-traumatic DOC and focusing only on one (the DMN) or few resting state networks when considering consciousness study. We avoided these biases looking at the whole brain with no prior assumption and considering strictly the subacute stage of sTBI. Consequently, we reported hyper- and hypo-connectivity in many regions of the brain but, as no single region accounted significantly for HDI change between clinical conditions, we did not identify ones from the DMN specifically explaining the whole brain reorganization. This suggests that brain reorganization is not solely explained by intra DMN connectivity changes in altered and recovered states of consciousness after subacute sTBI.

### The hub disruption index quantifies these patterns of simultaneous hyper- and hypo-connectivity in different states of consciousness

The HDI considers the simultaneous existence of topologically distributed hyper- and hypo-connectivities.^34^ Large hyper- and hypo-connectivities are translated to a large HDI absolute value while smaller hyper- and hypo-connectivities provide a smaller HDI absolute value. The HDI can be computed for all nodal metrics (Fig. 5): the HDI-degree used in this study corresponds to the disruption of integration properties of a graph, the HDI-clustering corresponds to the disruption of segregation properties whereas the HDI-BC corresponds to the disruption of nodal centrality properties. Providing long acquisition duration, as is done in the present study, the HDI has been shown to be a reliable index to quantify graph reorganization.^35^ Previous studies using nodal metrics highlighted brain network disruption in comatose^36^ and UWS/VS^34^ anoxic patients.

Taken together with our present study, these results suggest a gradient of severity in DOC with increasing HDI-degree and increasing HDI-clustering (in absolute values) (Table 4).

**Table 4:** HDI values according to the severity of DOC

HDI median	Comatose anoxic *n* = 25 (see Ref.^36^)	UWS/VS anoxic *n* = 17 (see Ref.^34^)	MCS TBI *n* = 11^a^	Conscious TBI *n* = 28^a^	Healthy subjects *n* = 20 (see Ref.^64^)
Clustering	−0.63	−0.75	−0.58	−0.26	0
Degree	−0.58	−0.82	−0.44	−0.2	0

^a^HDI values observed across previous studies according to the severity of DOC (despite not strictly comparable methodological steps between studies). HDI median for clustering and degree metrics are reported for different groups (*n* = number of subjects per group). HDI results of our present study are tagged.

DOC, disorder of consciousness; UWS, unresponsive wakefulness syndrome/vegetative state; MCS, minimally conscious state; C, conscious state.

### Relationship between brain network disruption and consciousness recovery

We showed that the HDI values, related to segregation and integration properties of the graph, are sensitive to consciousness state rather than time elapsed. While patients recover consciousness, the brain network topology evolves towards a physiological pattern of integration and segregation. The link between restoration of segregation network properties and consciousness recovery is an original result in sTBI, a pathology responsible for long range disruption secondary to axonal injuries. On a rodent TBI model inducing a sensorimotor cortical contusion and DAI, functional hypo-connectivity in the motor network and whole brain hyper-connectivity were both observed during the period of physical impairment. Interestingly in this animal TBI model, the anatomical recovery is well described, supported by axonal sprouting and cortical remapping, giving a biophysical support to the restoration of functional integration and segregation properties of the graph along with neurological recovery.^65^ Such preclinical data feed the hypothesis of a link between functional topological changes and cortical remapping during subacute sTBI in human. Indeed the role of functional hyper-connectivity is hypothesized to be a compensatory mechanism probably promoted by an enhanced segregation in brain network during the subacute stage^23^ that tends to decrease in chronic stage.^63,66–68^ We did not measure structural connectivity in our study, but regression of hyper-segregation in the structural connectome is documented during consciousness recovery in DOC,^69^ as well as during cognitive recovery in conscious TBI.^70,71^

Contrary to clustering and degree, the BC, which measures how central is a node in the brain network, was not sensitive to consciousness state in our study. Segregation and integration metrics have previously been reported more relevant than centrality ones to differentiate UWS/VS and MCS.^72^ As the patients of our cohort are still suffering cognitive impairments as shown by the disability rating scores, BC might be a metric sensitive to cognition rather than consciousness as reported also in chronic cognitively impaired TBI patients.^73^

### Methodological considerations and limitations of our study

The feasibility of rs-fMRI during subacute sTBI is questioned by the high proportion of data lost because of insufficient quality (40% in our study). Head motion was, as described elsewhere, the major cause of decreased signal to noise ratio in sTBI.^28,74,75^

A possible limitation of our approach is the presence of some large movements observed in patients. We carefully considered the movements using ART toolbox (Supplementary Fig. 3). The large deviation of movements was observed on short parts of time series, and as detailed in the method part, we removed the BOLD images impacted by the movements. We may also hypothesize that the wavelets are removing some effects of the large movements’ parameters. We finally controlled the level of BOLD variations for all the groups, and we were able to confirm that no significant difference in BOLD variations was observed (Supplementary Fig. 3).

Another limitation of the study could rely on the lesion locations that may differ between group of patients. We addressed this question and found no significant difference in the lesion location according to the group (see Supplementary material) suggesting that the observed differences in the brain topology of patients rather relate to the effect of consciousness.

Knowing the impact of sedation on resting state connectivity,^76^ we chose to strictly avoid it. The balance between benefit and risk of curare use under ventilatory support should be considered in the future. Also, the tracking of wakefulness during the acquisition should be considered as deep sleep reduces resting state intrinsic networks connectivity.^77,78^ No UWS/VS could be fully analysed in the cohort to corroborate the hypothesis that topological disruption of functional hubs is a correlate of consciousness in sTBI. This is a future goal for validation study. Nevertheless, the topological disintegration of hubness has been reported in coma.^36^

This work is derived from rs fMRI analysis that present a normal neurovascular coupling in HS. Such coupling might be impaired in sTBI. Because the hemodynamic response function can be estimated from resting-state acquisitions,^79^ we conducted an evaluation of this response in the DAI patient’s group (Supplementary Fig. 10). A single difference was found at group level in the white matter but none in the GM. Thus, potential alteration of the neurovascular coupling is probably not responsible for the clustering and degree properties alterations described in this work. Therefore, we considered the spontaneous fluctuations of the BOLD signal still relevant even in contused cortical areas, knowing that assumption is debated.^80^ If there is no BOLD signal inside an ROI damaged by a contusion, the graph attributes one single edge to this ROI and eventually reallocate the other edges of the considered ROI to other ROI of the network. Nevertheless, we checked the non-difference between DAI and DAI plus contusions in our cohort, making the impact of GM lesion on graph topological change less plausible. So, our results suggest that the HDI discriminates patients according to their state of consciousness despite the heterogeneity of the lesions.

## Conclusion

For the first time, extracting the HDI directly from each patient’s graph, we were able to track the clinical alteration and subsequent recovery of consciousness during the 3 months following a severe TBI. Graph analysis can implement a multimodal functional neuroimaging assessment in DOC and pave the way for a personalized medicine in severely brain-injured patients. The HDI diagnostic value for consciousness detection might therefore be further studied.

## Supplementary Material

fcad319_Supplementary_Data

## Data Availability

Patients’ data could be asked to the authors under reasonable request.
