# Supplementary material for: Functional hub disruption emphasizes consciousness recovery in severe traumatic brain injury
Source: Brain Commun. 2023 Nov 22;5(6):fcad319. doi: 10.1093/braincomms/fcad319 (PMC11098044; doi:10.1093/braincomms/fcad319)
Supplement: fcad319_Supplementary_Data [file fcad319_supplementary_data.docx]

**Supplementary Material**

**Disability measurement**

The Disability Rating Scale (DRS) consists of 8 items divided into four categories: arousal and awareness, cognitive ability to handle self-care functions, physical dependence upon others and psychosocial adaptability for work, housework, or school. The DRS score ranges from zero (no disability) to 29 (extreme vegetative state)^1^.

DRS scores were collected at Time 1 and Time 2 for each patient (Supplementary Table 1):

**TIME 1                                        TIME 2**

| Patients  ID | Delay between  rs-fMRI and sTBI (day) | State of  consciousness | DRS score |  | Delay between  rs-fMRI and sTBI (day) | State of  consciousness | DRS score |
| --- | --- | --- | --- | --- | --- | --- | --- |
| 1 | 90 | UWS/VS | 26 |  | 193 | UWS/VS | 25 |
| 2 | 30 | MCS | 22 |  | 62 | C | 14 |
| 3 | 27 | MCS | 22 |  | 105 | C | 13 |
| 4 | 24 | C | 16 |  | 73 | C | 10 |
| 5 | 71 | MCS | 22 |  | 151 | MCS | 14 |
| 6 | 47 | C | 9 |  | 89 | C | 8 |
| 7 | 26 | C | 4 |  | 89 | C | 1 |
| 8 | 20 | C | 10 |  | 83 | C | 1 |
| 9 | 37 | MCS | 21 |  | 75 | C | 9 |
| 10 | 27 | MCS | 21 |  | 34 | MCS | 21 |
| 11 | 13 | C | 6 |  | N/A | N/A | N/A |
| 12 | 58 | MCS | 21 |  | 85 | MCS | 21 |
| 13 | 19 | C | 15 |  | N/A | N/A | N/A |
| 14 | 51 | MCS | 25 |  | 104 | MCS | 22 |
| 15 | 44 | C | 3 |  | 66 | C | 2 |
| 16 | 32 | MCS | 23 |  | 46 | C | 19 |
| 17 | 38 | C | 18 |  | 87 | C | 7 |
| 18 | 69 | MCS | 22 |  | 113 | C | 19 |
| 19 | 48 | C | 13 |  | 67 | C | 13 |
| 20 | 43 | C | 13 |  | N/A | N/A | N/A |
| 21 | 23 | MCS | 22 |  | 34 | C | 22 |
| 22 | 61 | MCS | 22 |  | 117 | MCS | 19 |
| 23 | 33 | MCS | 22 |  | 54 | C | 14 |
| 24 | 32 | C | 16 |  | 54 | C | 6 |
| 25 | 28 | C | 9 |  | N/A | N/A | N/A |
| 26 | 32 | C | 18 |  | 54 | C | 9 |
| 27 | 21 | C | 9 |  | 70 | C | 2 |
| 28 | 25 | MCS | 22 |  | N/A | N/A | N/A |
| 29 | 31 | MCS | 22 |  | 58 | MCS | 21 |
| 30 | 37 | UWS/VS | 28 |  | N/A | N/A | N/A |
| 31 | 47 | MCS | 22 |  | 160 | C | 13 |
| 32 | 33 | MCS | 20 |  | N/A | N/A | N/A |
| 33 | 42 | C | 6 |  | N/A | N/A | N/A |
| 34 | 21 | C | 9 |  | N/A | N/A | N/A |

**Supplementary Table 1: disability level at time 1 and time 2**

Abbreviations: C= conscious, MCS=minimally conscious state, UWS/VS= unresponsive wakefulness syndrome/vegetative state, DRS= disability rating scale, N/A= not applicable

**Number of subjects**

In^2^, the HDI was – 0.8 ± 0.4 for anoxic patients. Our hypothesis was to observe a comparable HDI for MCS- sTBI group. Using a bilateral test (MCS versus Conscious sTBI at discharge from ICU) with a alpha risk 0.05 and a study power 80%, the inclusion of 17 patients per group would detect a reduction of 50% of the HDI value.

**MRI parameters**

The main parameters of the fMRI sequence were a gradient echo EPI sequence, 32 slices, 3.5 mm-thick, acquired with a multiband factor of two, in plane voxel siz=3x3 mm^2^, TR=2s, TE=32ms, flip-angle=75°, SENSE factor=3,5 dummy volumes and 400 volumes. The choice of TR=2s avoids contamination of the useful part of the signal (below 0.1 Hz) by participant’s breathing. Note that the slope of the commutation gradients was lowered to lower the scanner noise of the EPI sequence (SoftTone parameter = yes). The 3D MPRAGE parameters were 0.9x0.9x1.2 mm^3^, TI=0.8s, TR=3s, TE=28 ms, flip=15°, SENSE factor=4.4.

**Illustration of pre-processing results in case of cranioencephalic lesions**

**Example 1 (patient n°6):** The following example shows a contusion that could have been considered for applying a mask lesion while pre-processing rs fMRI data. Nevertheless, we correctly segmented the grey matter despite the lesion. Hydrocephalus (ventriculomegaly) was also correctly delimited with our preprocessing.

**A B**


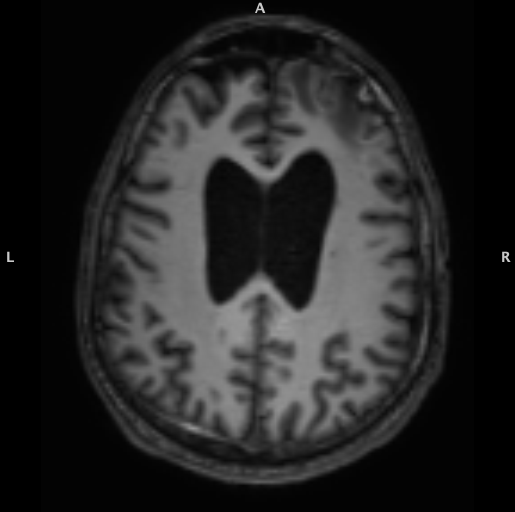

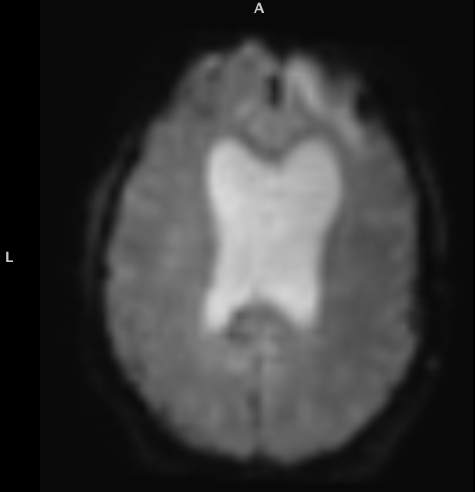


**C D**


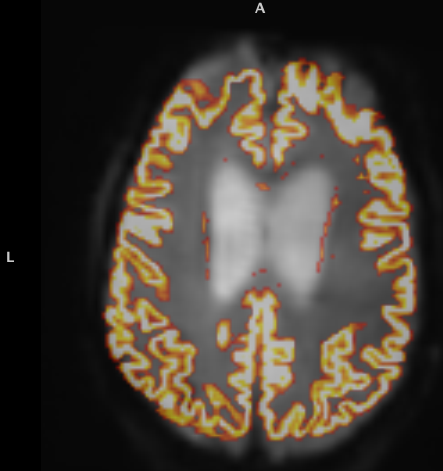

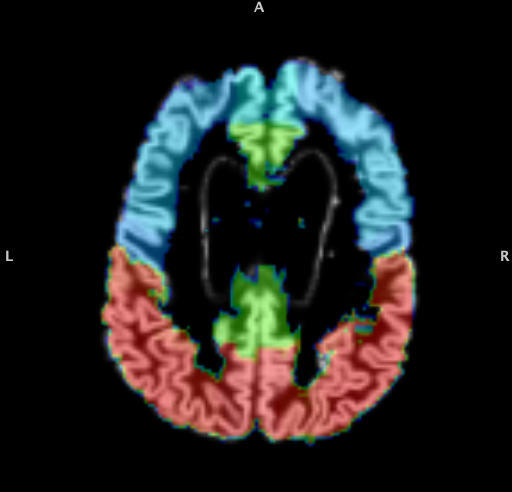


**Supplementary Figure 1**: **cortical contusion**

Panel A: tridimensional (3D) T1 MRI sequence, Panel B: functional MRI sequence, Panel C: functional MRI sequence co-registered with T13D gives the grey matter segmentation superimposed in warm color: right frontal cortico-subcortical contusion is considered for grey matter segmentation despite abnormal MRI signal, Panel D: customized AAL107 template superimposed (in spectrum color) on the normalized grey matter segmentation using DARTEL.

**Example 2 (patient n°19):** The following example shows the heterogeneity of the cranioencephalic lesions observed in our cohort that could not have been solved with a mask lesion: hydrocephalus and meningocele complicating a craniectomy. Our preprocessing successfully delimitates the grey matter.

**A B**


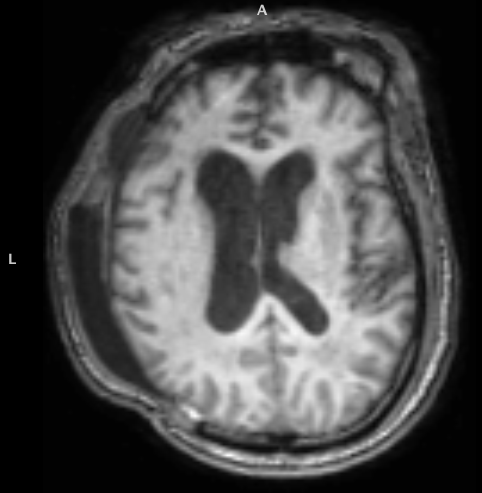

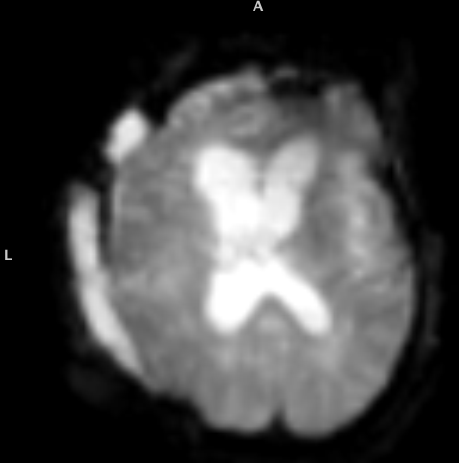


**C D**


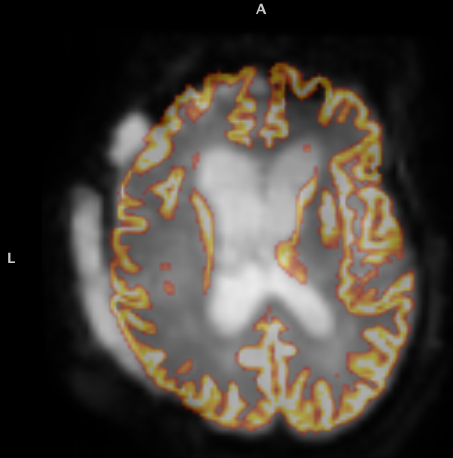

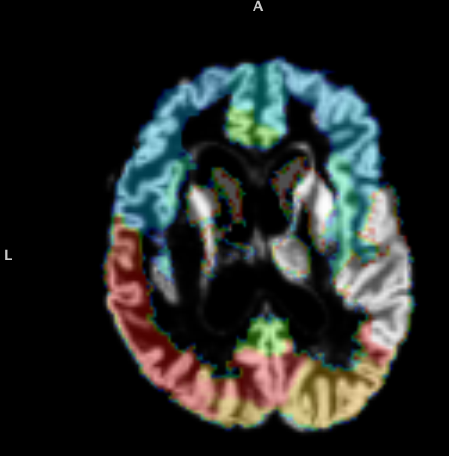


**Supplementary Figure 2: meningocele**

Panel A: MRI sequence tridimensional T1 showing a left meningocele (subdural cerebrospinal fluid collection) in a case of left craniectomy for a severe TBI, Panel B: functional MRI sequence, Panel C: functional MRI sequence co-registered with T1 tridimensional sequence gives the grey matter segmentation superimposed in warm color: left meningocele under a craniectomy does not impair co-registration of grey matter. Hydrocephalus (ventriculomegaly) was also correctly delimited with our preprocessing, Panel D: customized AAL107 template superimposed (in spectrum color) on the normalized grey matter segmentation using DARTEL.

**AAL 107 template**

In the following Supplementary Table 2 we listed the 107 grey matter brain regions we extracted from the AAL116 template, their voxel size and volume.

L: left, R: right

| **AAL 107 template extracted from the AAL116** | | |
| --- | --- | --- |
| **Regions** | **number of voxel** | **volume in mm^3^** |
| Precentral_L | 3526 | 28208 |
| Precentral_R | 3381 | 27048 |
| Frontal_Sup_2_L | 4870 | 38960 |
| Frontal_Sup_2_R | 5126 | 41008 |
| Frontal_Mid_2_L | 4507 | 36056 |
| Frontal_Mid_2_R | 4860 | 38880 |
| Frontal_Inf_Oper_L | 1038 | 8304 |
| Frontal_Inf_Oper_R | 1399 | 11192 |
| Frontal_Inf_Tri_L | 2529 | 20232 |
| Frontal_Inf_Tri_R | 2151 | 17208 |
| Frontal_Inf_Orb_2_L | 814 | 6512 |
| Frontal_Inf_Orb_2_R | 874 | 6992 |
| Rolandic_Oper_L | 988 | 7904 |
| Rolandic_Oper_R | 1331 | 10648 |
| Supp_Motor_Area_L | 2147 | 17176 |
| Supp_Motor_Area_R | 2371 | 18968 |
| Olfactory_L | 291 | 2328 |
| Olfactory_R | 288 | 2304 |
| Frontal_Sup_Medial_L | 2992 | 23936 |
| Frontal_Sup_Medial_R | 2134 | 17072 |
| Frontal_Med_Orb_L | 719 | 5752 |
| Frontal_Med_Orb_R | 856 | 6848 |
| Rectus_L | 852 | 6816 |
| Rectus_R | 745 | 5960 |
| OFCmed_L | 550 | 4400 |
| OFCmed_R | 621 | 4968 |
| OFCant_L | 443 | 3544 |
| OFCant_R | 648 | 5184 |
| OFCpost_L | 567 | 4536 |
| OFCpost_R | 561 | 4488 |
| OFClat_L | 197 | 1576 |
| OFClat_R | 188 | 1504 |
| Insula_L | 1858 | 14864 |
| Insula_R | 1770 | 14160 |
| Cingulate_Mid_L | 1941 | 15528 |
| Cingulate_Mid_R | 2203 | 17624 |
| Cingulate_Post_L | 463 | 3704 |
| Cingulate_Post_R | 335 | 2680 |
| Hippocampus_L | 932 | 7456 |
| Hippocampus_R | 946 | 7568 |
| ParaHippocampal_L | 978 | 7824 |
| ParaHippocampal_R | 1132 | 9056 |
| Amygdala_L | 220 | 1760 |
| Amygdala_R | 248 | 1984 |
| Calcarine_L | 2258 | 18064 |
| Calcarine_R | 1861 | 14888 |
| Cuneus_L | 1521 | 12168 |
| Cuneus_R | 1424 | 11392 |
| Lingual_L | 2095 | 16760 |
| Lingual_R | 2300 | 18400 |
| Occipital_Sup_L | 1366 | 10928 |
| Occipital_Sup_R | 1413 | 11304 |
| Occipital_Mid_L | 3265 | 26120 |
| Occipital_Mid_R | 2098 | 16784 |
| Occipital_Inf_L | 941 | 7528 |
| Occipital_Inf_R | 989 | 7912 |
| Fusiform_L | 2309 | 18472 |
| Fusiform_R | 2518 | 20144 |
| Postcentral_L | 3892 | 31136 |
| Postcentral_R | 3823 | 30584 |
| Parietal_Sup_L | 2065 | 16520 |
| Parietal_Sup_R | 2222 | 17776 |
| Parietal_Inf_L | 2447 | 19576 |
| Parietal_Inf_R | 1345 | 10760 |
| SupraMarginal_L | 1256 | 10048 |
| SupraMarginal_R | 1974 | 15792 |
| Angular_L | 1173 | 9384 |
| Angular_R | 1752 | 14016 |
| Precuneus_L | 3528 | 28224 |
| Precuneus_R | 3265 | 26120 |
| Paracentral_Lobule_L | 1349 | 10792 |
| Paracentral_Lobule_R | 836 | 6688 |
| Caudate_L | 805 | 6440 |
| Caudate_R | 861 | 6888 |
| Putamen_L | 999 | 7992 |
| Putamen_R | 1062 | 8496 |
| Pallidum_L | 293 | 2344 |
| Pallidum_R | 280 | 2240 |
| Heschl_L | 225 | 1800 |
| Heschl_R | 249 | 1992 |
| Temporal_Sup_L | 2296 | 18368 |
| Temporal_Sup_R | 3141 | 25128 |
| Temporal_Pole_Sup_L | 1285 | 10280 |
| Temporal_Pole_Sup_R | 1338 | 10704 |
| Temporal_Mid_L | 4942 | 39536 |
| Temporal_Mid_R | 4409 | 35272 |
| Temporal_Pole_Mid_L | 755 | 6040 |
| Temporal_Pole_Mid_R | 1187 | 9496 |
| Temporal_Inf_L | 3200 | 25600 |
| Temporal_Inf_R | 3557 | 28456 |
| Thalamus_lateral_L | 4480 | 2090 |
| Thalamus_lateral_R | 4400 | 2090 |
| Thalamus_medial_L | 1760 | 888 |
| Thalamus_medial_R | 1800 | 888 |
| Thalamus_pul_L | 2248 | 1196 |
| Thalamus_pul_R | 2176 | 1196 |
| ACC_sub_L | 168 | 1344 |
| ACC_sub_R | 132 | 1056 |
| ACC_pre_L | 627 | 5016 |
| ACC_pre_R | 648 | 5184 |
| ACC_sup_L | 605 | 4840 |
| ACC_sup_R | 533 | 4264 |
| Cerebelum_L | 1261 | 10088 |
| Cerebelum_R | 1068 | 8544 |
| Vermis | 101 | 1316 |
| Cerebelum_Crus_L | 6776 | 54208 |
| Cerebelum_Crus_R | 7094 | 56752 |

**Supplementary Table 2: Template AAL107**

**Head movement regression: spatial standard deviation of successive difference images (DVARS) and Framewise Displacement (FD)**

We plotted in the following diagrams for each group (minimally conscious TBI (MCS), conscious TBI (C) and healthy subjects (HS)) the 3-dimensional parameters of head movements during rs fMRI acquisitions.

We recomputed two parameters for quantifying movement effect: Framewise displacement (FD) on the movement parameters extracted directly from pre-processing and DVARS (corresponding to the spatial root mean square of the data after temporal differencing) on the pre-processed time series. We applied a multivariate test by comparing average of the parameters computed at each frame using (Power et al. 2012) approach^3^.

We found that FD is significantly different across the 3 groups: HS, C and MCS. However, DVARS is not significantly different.

We also applied paired tests using Wilcoxon tests, and significant differences were obtained using FD, but no significant differences were observed using DVARS. We also plotted the same representation as proposed in (Power et al. 2012) representing the variations in BOLD in comparisons to FD (not shown). In these plots, no effect of FD on the variations in BOLD were observed. That is, the displacements had no impact on BOLD measure after pre-processing. This might be due to the way we apply pre-processing using ART (rejection of artefacted images) and wavelets transforms (removal of frequencies out of the frequency range of interest). Doing so we were able to remove the possible dramatic effect of movements on time-course correlations.

DVARS FD

**Supplementary Figure 3:** Motion parameters DVARS (left) and FD (right) according to the three groups. FD are significantly different among groups while the DVARS are not. Statistical tests are Mann-Whitney tests for paired comparison and significance level is consequently indicated by stars: * = 0.05 ** = 0.005 *** = 0.0005 while NS indicates non-significant test. Then a Kruskal-Wallis test is applied for multiple group comparison and the corresponding p-value is provided.

**Lesion probability map for each group**

In order to assess whether lesion maps could play a differential role according to group status, TBI related lesions were compared between MCS and C groups. Since concussion contains oedema and bleeding, iron level is enhanced in the lesion resulting in distortion in the magnetic field and reduction of BOLD signal. To map these lesions, we thus computed the maps of hyposignals observed in the mean functional image of each patient (each mean functional image after rejection of artefacted images was warped in the MNI referential applying DARTEL deformation field). A mask of within-brain hyposignals was computed per subject and exam (and checked by visual inspection) using a homemade program under Matlab. For each group of patients, an averaged hyposignal lesion image was derived. To compare those lesion images, a two-sample t-test was performed to reject or not null hypothesis. No significant difference was found between both groups of patients, suggesting that the between group differences observed between graph properties are not related to a given location of lesions.

**Graph metrics**

Description of the network metrics is provided in Supplementary Table 2. Detailed information and metrics computation can be found in^4^. We explored both regional metrics computed at the level of the nodes of the graphs and global metrics that correspond to the average of the regional metrics other the whole graph.

| **Graph metrics** | **Character** | **Description** |
| --- | --- | --- |
| **Regional networks metrics** | | |
| Global efficiency | E_glob(i)_ | Communication efficiency of a node (i) with all other nodes |
| Degree | Deg_(i)_ | Number of edges connected to a node (i) |
| Clustering  (also named local efficiency) | Clustering_(i)_ | Communication efficiency between all first neighbours of a node (i) |
| Clustering coefficient | CC_(i)_ | Fraction of the neighbours of a node (i) that are also neighbours of each others |
| Betweenness centrality | BC_(i)_ | Influence of a node (i) over the minimum path of all pairs of nodes |
| **Global network metrics** | | |
| Modularity | Q | Strength of division of a network into clusters of nodes highly connected between them |
| Global efficiency | E_glob_ | Mean of  E_glob(i)_ |
| Clustering  (also named local efficiency) | Clustering | Mean of Clustering_(i)_ |
| Clustering coefficient | CC | Mean of  CC_(i)_ |
| Betweenness centrality | BC | Mean of BC_(i)_ |

**Supplementary Table 3: graph metrics description**

**Means for each graph metric**

The following supplementary figures 4 to 6 show the results for each metric and each group at time 1 for all TBI patients compared to healthy subjects (Supplementary fig. 4), at time 2 for all TBI patients compared to healthy subjects (Supplementary fig. 5) and at time 1 for each clinical condition (conscious and minimally conscious) compared to healthy subjects (Supplementary fig. 6). No significant difference was observed between healthy subjects and patients at time 1 and 2 except for modularity. No significant difference between clinical conditions was observed except for global efficiency at time 1.

**Supplementary Figure 4: Mean values for each metric at Time 1.**

Eglob: global efficiency, Eloc: local efficiency (= clustering), BC: betweenness centrality.

Control= healthy subjects.

All statistical tests are Mann-Whitney tests. Significance level is indicated by stars: * = 0.05 ** = 0.005 *** = 0.0005. NS indicates non-significant test.

**Supplementary Figure 5: Time 2**

Eglob: global efficiency, Eloc: local efficiency (= clustering), BC: betweenness centrality.

Control= healthy subjects.

All statistical tests are Mann-Whitney tests. Significance level is indicated by stars: * = 0.05 ** = 0.005 *** = 0.0005. NS indicates non-significant test.

**Supplementary Figure 6: Time 1 for each clinical group and controls**

Eglob: global efficiency, Eloc: local efficiency (= clustering), BC: betweenness centrality.

HS= healthy subjects, C= conscious sTBI, MCS: minimally conscious sTBI

Statistical tests are Mann-Whitney tests for paired comparison and significance level is consequently indicated by stars: * = 0.05 ** = 0.005 *** = 0.0005 while NS indicates non-significant test. Then a Kruskal-Wallis test is applied for multiple group comparison and the corresponding p-value is provided.

**Mixed effect model**

The linear multivariable mixed-effects regression model with random intercept corresponds to the following equation:

$$HDI\left( ij \right)=\beta0+\beta1*Consciousness\left( ij \right)+\beta2*Time\left( j \right)+\beta3*Consciousness\left( ij \right)\times Time\left( j \right)+\beta\left( 0i \right)+\epsilon\left( i \right)$$

In this model, the predicted HDI of interest for subject i (HDI i) is determined by population (fixed) effects, represented by $\beta0$, $\beta$1, $\beta$2, $\beta$3 and subject-specific (random) effects, $\beta$0i. The average rate of change in the population is $\beta$1, and the subject-specific difference in that rate is captured by $\beta$0i. Time was modeled as an indicator variable, Time 1 and Time 2. Interaction between conscious state and time was also included in the model. Our variable of interest is Consciousness, we modelled it as an indicator function taking two values: MCS and C. Statistical analysis was performed using R version 4.1.0 (Core Team (2018). R: A language and environment for statistical computing. R Foundation for Statistical Computing, Vienna, Austria. URL https://www.R-project.org/). The blme package version was used for the fitting of mixed effects models using restricted maximum likelihood estimation.

The supplementary Table 4 reports the coefficient, 95% confidence interval and p value for the variables included in the mixed effect model (intercept, consciousness, time, consciousness and time interaction) considering the HDI betweenness centrality and the modularity.

|  | | | |
| --- | --- | --- | --- |
| $HDI=effects+effects*Consciousness+effects*Time+effects*Consciousness\times Time+randomintercept+randomeffect$ | | | |
| HDI BC | Coefficient | 95% CI | P value |
| Intercept | -0.50 | [-0.65, -0.33] | <0.01 |
| Consciousness | -0.20 | [-0.46, 0.03] | 0.104 |
| Time | 0.006 | [-0.18, 0.18] | 0.95 |
| Consciousness and Time interaction | 0.10 | [-0.45, 0.67] | 0.698 |
|  |  |  |  |
|  |  |  |  |
|  |  |  |  |
| MODULARITY |  |  |  |
| Intercept | 0.57 | [0.54, 0.61] | < .001 |
| Consciousness | -0.05 | [-0.10, 0.01] | 0.110 |
| Time | 0.004 | [-0.04, 0.04] | 0.881 |
| Consciousness and Time interaction | -0.008 | [-0.12, 0.11] | 0.887 |

**Supplementary Table 4: impact of time and consciousness explored with a mixed effect model, results for the hub disruption index (HDI) considering betweenness centrality (BC) and for modularity**

**Regions of interest with significant p values, group comparison**

Here we listed for each region of interest the p value obtained when applying a Wilcoxon rank test to explore if one or several regions accounted for the HDI change between clinical condition and between groups. This was done for the 4 main metrics calculated in the graph.

For each table (Supplementary Tables 5 to 7), the ROI have been ordered from the lower to the higher pvalue considering **clustering**:

**Supplementary Table 5: MCS versus healthy subjects**

| ROI | Eglob | **Clustering** | BC | Degree |
| --- | --- | --- | --- | --- |
| Occipital_Inf_L | 0,030 | 0,000 | 0,520 | 0,010 |
| Cuneus_R | 0,033 | 0,000 | 0,001 | 0,802 |
| Fusiform_L | 0,010 | 0,000 | 0,012 | 0,000 |
| Occipital_Mid_L | 0,496 | 0,000 | 0,496 | 0,011 |
| Fusiform_R | 0,103 | 0,001 | 0,757 | 0,000 |
| Occipital_Mid_R | 0,788 | 0,001 | 0,030 | 0,029 |
| Heschl_L | 0,079 | 0,002 | 0,298 | 0,018 |
| Putamen_L | 0,027 | 0,005 | 0,915 | 0,550 |
| Olfactory_L | 0,332 | 0,005 | 0,926 | 0,524 |
| Occipital_Sup_L | 0,853 | 0,009 | 0,231 | 0,007 |
| Frontal_Mid_2_L | 0,256 | 0,009 | 0,231 | 0,110 |
| Insula_R | 0,223 | 0,014 | 0,549 | 0,147 |
| Rolandic_Oper_L | 0,984 | 0,015 | 0,917 | 0,178 |
| Pallidum_L | 0,073 | 0,018 | 0,402 | 0,141 |
| OFCmed_R | 0,788 | 0,020 | 0,073 | 0,065 |
| Occipital_Sup_R | 0,788 | 0,021 | 0,024 | 0,017 |
| ParaHippocampal_L | 0,079 | 0,022 | 0,982 | 0,143 |
| OFCmed_L | 0,060 | 0,024 | 0,323 | 0,128 |
| Parietal_Inf_R | 0,292 | 0,026 | 0,186 | 0,661 |
| OFClat_L | 0,223 | 0,026 | 0,274 | 0,090 |
| Olfactory_R | 0,180 | 0,028 | 0,564 | 0,141 |
| Frontal_Sup_2_R | 0,332 | 0,029 | 0,726 | 0,085 |
| Frontal_Mid_2_R | 0,274 | 0,030 | 0,726 | 0,161 |
| Rectus_L | 0,055 | 0,036 | 0,039 | 0,009 |
| Cuneus_L | 0,095 | 0,046 | 0,369 | 0,519 |
| Lingual_L | 0,496 | 0,050 | 0,726 | 0,004 |
| OFCant_L | 0,606 | 0,059 | 0,945 | 0,853 |
| Putamen_R | 0,397 | 0,059 | 0,120 | 0,590 |
| ACC_sub_L | 0,027 | 0,059 | 1,000 | 0,871 |
| Pallidum_R | 0,421 | 0,059 | 0,572 | 0,047 |
| Temporal_Sup_L | 0,122 | 0,059 | 0,444 | 0,016 |
| Precuneus_L | 0,073 | 0,060 | 0,312 | 0,030 |
| Postcentral_R | 0,000 | 0,060 | 0,009 | 0,006 |
| Temporal_Inf_R | 0,022 | 0,066 | 0,470 | 0,012 |
| Rectus_R | 0,066 | 0,067 | 0,036 | 0,012 |
| Precuneus_R | 0,016 | 0,069 | 0,353 | 0,005 |
| Temporal_Inf_L | 0,193 | 0,074 | 0,917 | 0,183 |
| Insula_L | 0,397 | 0,077 | 0,374 | 0,152 |
| Thalamus_medial_R | 0,820 | 0,082 | 0,079 | 0,009 |
| Lingual_R | 0,397 | 0,083 | 0,951 | 0,000 |
| Occipital_Inf_R | 0,087 | 0,086 | 0,884 | 0,000 |
| OFClat_R | 0,193 | 0,091 | 0,636 | 0,248 |
| Amygdala_R | 0,193 | 0,094 | 0,696 | 0,569 |
| Rolandic_Oper_R | 0,208 | 0,098 | 0,041 | 0,967 |
| Frontal_Inf_Tri_R | 0,577 | 0,105 | 0,037 | 0,632 |
| Thalamus_lateral_L | 0,050 | 0,110 | 0,095 | 0,045 |
| SupraMarginal_R | 0,143 | 0,111 | 0,154 | 0,723 |
| Frontal_Sup_Medial_L | 0,005 | 0,111 | 0,536 | 0,095 |
| Precentral_L | 0,037 | 0,117 | 0,020 | 0,116 |
| Caudate_L | 0,312 | 0,142 | 0,946 | 0,771 |
| Frontal_Inf_Orb_2_R | 0,112 | 0,160 | 0,080 | 0,144 |
| Frontal_Inf_Oper_R | 0,193 | 0,164 | 0,481 | 0,755 |
| Temporal_Pole_Mid_R | 0,549 | 0,166 | 0,590 | 0,215 |
| ParaHippocampal_R | 0,132 | 0,171 | 0,604 | 0,207 |
| Parietal_Sup_L | 0,496 | 0,173 | 0,143 | 1,000 |
| ACC_sub_R | 0,073 | 0,200 | 0,116 | 0,069 |
| Hippocampus_R | 0,180 | 0,205 | 0,982 | 0,384 |
| Calcarine_L | 1,000 | 0,238 | 0,901 | 0,394 |
| Calcarine_R | 0,154 | 0,263 | 0,739 | 0,018 |
| Heschl_R | 0,397 | 0,293 | 0,088 | 0,095 |
| Frontal_Sup_Medial_R | 0,256 | 0,330 | 0,509 | 0,157 |
| Frontal_Inf_Orb_2_L | 0,984 | 0,334 | 0,525 | 0,432 |
| Frontal_Sup_2_L | 0,055 | 0,342 | 0,853 | 0,029 |
| OFCpost_R | 0,918 | 0,348 | 0,462 | 0,650 |
| Thalamus_pul_L | 0,024 | 0,354 | 0,187 | 0,101 |
| ACC_sup_R | 0,375 | 0,362 | 0,348 | 0,738 |
| Postcentral_L | 0,001 | 0,364 | 0,154 | 0,030 |
| Cerebelum_R | 0,239 | 0,372 | 0,425 | 0,688 |
| Paracentral_Lobule_L | 0,066 | 0,373 | 0,575 | 0,253 |
| Paracentral_Lobule_R | 0,312 | 0,383 | 0,900 | 0,663 |
| ACC_pre_R | 0,577 | 0,394 | 0,802 | 0,851 |
| Cingulate_Post_R | 0,143 | 0,400 | 0,816 | 0,850 |
| Angular_R | 0,008 | 0,403 | 0,123 | 0,030 |
| Temporal_Mid_R | 0,292 | 0,407 | 0,011 | 0,129 |
| Cingulate_Post_L | 0,421 | 0,412 | 0,159 | 0,674 |
| Cingulate_Mid_L | 0,256 | 0,432 | 0,045 | 0,192 |
| OFCant_R | 0,635 | 0,454 | 0,191 | 0,258 |
| Cerebelum_L | 0,332 | 0,484 | 1,000 | 0,297 |
| Temporal_Pole_Mid_L | 0,193 | 0,491 | 0,044 | 0,085 |
| Thalamus_lateral_R | 0,167 | 0,519 | 0,817 | 0,896 |
| Thalamus_pul_R | 0,020 | 0,571 | 0,109 | 0,166 |
| Supp_Motor_Area_L | 0,239 | 0,605 | 0,820 | 0,270 |
| Angular_L | 0,397 | 0,641 | 0,053 | 0,218 |
| SupraMarginal_L | 0,073 | 0,642 | 0,724 | 0,243 |
| Temporal_Pole_Sup_R | 0,665 | 0,645 | 0,032 | 0,165 |
| Cerebelum_Crus_R | 0,132 | 0,649 | 0,060 | 0,147 |
| ACC_sup_L | 0,635 | 0,675 | 0,404 | 0,369 |
| Temporal_Sup_R | 0,112 | 0,692 | 0,030 | 0,029 |
| Frontal_Med_Orb_L | 0,154 | 0,717 | 0,068 | 0,364 |
| Frontal_Med_Orb_R | 0,522 | 0,726 | 0,535 | 0,188 |
| Parietal_Inf_L | 0,180 | 0,739 | 0,549 | 0,263 |
| ACC_pre_L | 0,312 | 0,750 | 0,374 | 0,900 |
| Supp_Motor_Area_R | 0,397 | 0,788 | 0,522 | 0,694 |
| Frontal_Inf_Tri_L | 0,820 | 0,802 | 0,119 | 0,755 |
| Caudate_R | 0,885 | 0,834 | 0,794 | 0,709 |
| Temporal_Pole_Sup_L | 0,208 | 0,859 | 0,003 | 0,046 |
| Hippocampus_L | 0,132 | 0,872 | 0,640 | 0,861 |
| Frontal_Inf_Oper_L | 0,332 | 0,882 | 0,983 | 0,613 |
| Temporal_Mid_L | 0,757 | 0,917 | 0,804 | 0,709 |
| Parietal_Sup_R | 0,665 | 0,934 | 0,984 | 0,468 |
| Vermis | 0,055 | 0,936 | 0,509 | 0,230 |
| Precentral_R | 0,037 | 0,951 | 0,757 | 0,089 |
| OFCpost_L | 0,820 | 0,959 | 0,945 | 0,930 |
| Thalamus_medial_L | 0,050 | 0,980 | 0,136 | 0,511 |
| Cingulate_Mid_R | 0,470 | 1,000 | 0,726 | 0,507 |
| Cerebelum_Crus_L | 0,079 | 1,000 | 0,013 | 0,051 |
| Amygdala_L | 0,132 | 1,000 | 0,201 | 0,174 |

**Supplementary Table 6: Conscious versus healthy subjects**

| ROI | Eglob | **Clustering** | BC | Degree |
| --- | --- | --- | --- | --- |
| Fusiform_R | 0,171 | 0,000 | 0,046 | 0,018 |
| Fusiform_L | 0,191 | 0,001 | 0,331 | 0,006 |
| Cingulate_Mid_L | 0,229 | 0,004 | 0,310 | 0,197 |
| OFClat_L | 0,272 | 0,007 | 0,036 | 0,004 |
| Rolandic_Oper_R | 0,092 | 0,008 | 0,017 | 0,275 |
| Occipital_Inf_L | 0,341 | 0,009 | 0,456 | 0,045 |
| Precuneus_R | 0,263 | 0,013 | 0,315 | 0,085 |
| Cuneus_R | 0,762 | 0,015 | 0,040 | 0,582 |
| Frontal_Mid_2_R | 0,272 | 0,017 | 0,623 | 0,140 |
| OFCant_L | 0,892 | 0,018 | 0,190 | 0,066 |
| Precuneus_L | 0,101 | 0,019 | 0,730 | 0,023 |
| Heschl_L | 0,164 | 0,039 | 0,474 | 0,021 |
| Putamen_R | 0,471 | 0,051 | 0,419 | 0,643 |
| Putamen_L | 0,021 | 0,051 | 0,798 | 0,516 |
| Temporal_Pole_Sup_R | 0,341 | 0,062 | 0,384 | 0,145 |
| Postcentral_L | 0,003 | 0,066 | 0,023 | 0,031 |
| OFCmed_L | 0,140 | 0,066 | 0,109 | 0,019 |
| Occipital_Mid_L | 0,246 | 0,088 | 0,616 | 0,013 |
| Occipital_Sup_R | 0,221 | 0,088 | 0,044 | 0,003 |
| Parietal_Sup_L | 0,942 | 0,092 | 0,433 | 0,047 |
| Paracentral_Lobule_L | 0,081 | 0,107 | 0,290 | 0,350 |
| Calcarine_R | 0,843 | 0,109 | 0,127 | 0,891 |
| Frontal_Mid_2_L | 0,385 | 0,126 | 0,608 | 0,138 |
| Frontal_Sup_2_R | 0,191 | 0,127 | 0,714 | 0,193 |
| ACC_sub_L | 0,551 | 0,142 | 0,283 | 0,334 |
| Occipital_Mid_R | 0,281 | 0,143 | 0,184 | 0,033 |
| Cerebelum_Crus_R | 0,013 | 0,145 | 0,017 | 0,001 |
| Parietal_Inf_R | 0,119 | 0,172 | 1,000 | 0,154 |
| Frontal_Inf_Oper_R | 0,177 | 0,179 | 0,691 | 0,598 |
| Rolandic_Oper_L | 0,081 | 0,198 | 0,119 | 0,825 |
| Vermis | 0,385 | 0,203 | 0,238 | 0,224 |
| ACC_sup_R | 0,992 | 0,205 | 0,670 | 0,625 |
| Caudate_R | 0,048 | 0,216 | 0,608 | 0,786 |
| Rectus_L | 0,048 | 0,223 | 0,006 | 0,005 |
| Olfactory_L | 0,537 | 0,239 | 0,488 | 0,636 |
| ACC_sub_R | 0,714 | 0,239 | 0,555 | 0,518 |
| Precentral_L | 0,025 | 0,246 | 0,061 | 0,077 |
| OFCmed_R | 0,458 | 0,278 | 0,715 | 0,497 |
| Postcentral_R | 0,032 | 0,290 | 0,154 | 0,155 |
| Calcarine_L | 0,254 | 0,295 | 0,187 | 0,628 |
| Frontal_Inf_Tri_R | 0,875 | 0,295 | 0,028 | 0,070 |
| Cingulate_Mid_R | 0,229 | 0,301 | 0,925 | 0,080 |
| Cerebelum_Crus_L | 0,070 | 0,302 | 0,094 | 0,069 |
| Precentral_R | 0,385 | 0,305 | 0,859 | 0,958 |
| Angular_R | 0,026 | 0,312 | 0,924 | 0,161 |
| Insula_L | 0,653 | 0,312 | 0,761 | 0,169 |
| Temporal_Sup_R | 0,074 | 0,327 | 0,036 | 0,016 |
| Parietal_Inf_L | 0,497 | 0,328 | 0,294 | 0,768 |
| Thalamus_lateral_L | 0,826 | 0,332 | 0,964 | 0,739 |
| ParaHippocampal_L | 0,246 | 0,344 | 0,846 | 0,900 |
| Paracentral_Lobule_R | 0,164 | 0,356 | 0,916 | 0,557 |
| Angular_L | 0,221 | 0,376 | 0,742 | 0,656 |
| Frontal_Med_Orb_L | 0,668 | 0,381 | 0,336 | 0,542 |
| Lingual_R | 0,421 | 0,397 | 0,714 | 0,071 |
| Thalamus_lateral_R | 0,551 | 0,416 | 0,068 | 0,071 |
| Occipital_Inf_R | 0,067 | 0,418 | 0,313 | 0,001 |
| Pallidum_L | 0,026 | 0,422 | 0,784 | 0,735 |
| Pallidum_R | 0,794 | 0,422 | 0,157 | 0,346 |
| Thalamus_medial_L | 0,608 | 0,449 | 0,630 | 0,328 |
| Frontal_Sup_Medial_R | 0,044 | 0,489 | 0,859 | 0,058 |
| Heschl_R | 0,321 | 0,491 | 0,005 | 0,062 |
| Supp_Motor_Area_L | 0,048 | 0,517 | 0,191 | 0,007 |
| OFCpost_R | 0,484 | 0,530 | 0,750 | 0,729 |
| Frontal_Med_Orb_R | 0,409 | 0,559 | 0,051 | 0,074 |
| OFCant_R | 0,301 | 0,559 | 0,259 | 0,360 |
| Frontal_Inf_Orb_2_R | 0,310 | 0,588 | 0,597 | 0,795 |
| Temporal_Pole_Sup_L | 0,056 | 0,607 | 0,010 | 0,055 |
| Frontal_Inf_Orb_2_L | 0,537 | 0,610 | 0,640 | 0,974 |
| Temporal_Pole_Mid_L | 0,291 | 0,649 | 0,426 | 0,629 |
| Cingulate_Post_L | 0,191 | 0,660 | 0,178 | 0,398 |
| Parietal_Sup_R | 0,892 | 0,668 | 0,523 | 0,128 |
| Caudate_L | 0,101 | 0,679 | 0,819 | 0,696 |
| Temporal_Mid_R | 0,958 | 0,699 | 0,259 | 0,875 |
| Cerebelum_L | 0,762 | 0,700 | 0,599 | 0,871 |
| Occipital_Sup_L | 0,206 | 0,707 | 0,884 | 0,005 |
| Thalamus_medial_R | 0,058 | 0,713 | 0,000 | 0,000 |
| Supp_Motor_Area_R | 0,152 | 0,714 | 1,000 | 0,116 |
| Temporal_Pole_Mid_R | 0,762 | 0,744 | 0,371 | 0,348 |
| ACC_sup_L | 0,433 | 0,750 | 1,000 | 0,547 |
| Amygdala_R | 0,762 | 0,760 | 0,052 | 0,119 |
| Temporal_Inf_L | 0,810 | 0,761 | 0,925 | 0,621 |
| Lingual_L | 0,668 | 0,762 | 0,983 | 0,165 |
| SupraMarginal_R | 0,746 | 0,776 | 0,245 | 0,866 |
| Olfactory_R | 0,778 | 0,784 | 1,000 | 0,749 |
| Thalamus_pul_L | 0,140 | 0,809 | 0,046 | 0,088 |
| Amygdala_L | 0,942 | 0,810 | 0,213 | 0,395 |
| Frontal_Sup_Medial_L | 0,013 | 0,818 | 0,623 | 0,064 |
| ParaHippocampal_R | 0,272 | 0,820 | 0,264 | 0,263 |
| Temporal_Sup_L | 0,445 | 0,826 | 0,061 | 0,151 |
| Insula_R | 0,925 | 0,850 | 0,272 | 0,867 |
| Cingulate_Post_R | 0,152 | 0,856 | 0,638 | 0,932 |
| Thalamus_pul_R | 0,030 | 0,862 | 0,154 | 0,435 |
| SupraMarginal_L | 0,146 | 0,864 | 0,793 | 0,241 |
| OFCpost_L | 0,875 | 0,866 | 0,393 | 0,667 |
| Hippocampus_R | 0,975 | 0,872 | 0,381 | 0,523 |
| Frontal_Inf_Tri_L | 0,537 | 0,883 | 0,057 | 0,992 |
| Frontal_Sup_2_L | 0,433 | 0,900 | 0,826 | 0,150 |
| ACC_pre_R | 0,135 | 0,905 | 0,240 | 0,703 |
| Temporal_Inf_R | 0,084 | 0,908 | 0,064 | 0,027 |
| Temporal_Mid_L | 0,246 | 0,916 | 0,638 | 0,274 |
| OFClat_R | 0,892 | 0,923 | 0,006 | 0,055 |
| Frontal_Inf_Oper_L | 0,025 | 0,941 | 0,730 | 0,158 |
| Rectus_R | 0,992 | 0,953 | 0,879 | 0,597 |
| Cerebelum_R | 0,875 | 0,958 | 0,267 | 0,505 |
| Hippocampus_L | 0,497 | 0,973 | 0,874 | 1,000 |
| Cuneus_L | 0,908 | 0,975 | 0,547 | 0,689 |
| ACC_pre_L | 0,310 | 1,000 | 0,133 | 0,751 |

**Supplementary Table 7: MCS versus Conscious sTBI**

| ROI | Eglob | **Clustering** | BC | Degree |
| --- | --- | --- | --- | --- |
| ParaHippocampal_L | 0,055 | 0,001 | 0,960 | 0,115 |
| Occipital_Inf_L | 0,156 | 0,005 | 0,863 | 0,099 |
| Occipital_Mid_L | 0,988 | 0,005 | 0,839 | 0,194 |
| Cuneus_L | 0,115 | 0,007 | 0,106 | 0,604 |
| Thalamus_lateral_L | 0,010 | 0,010 | 0,075 | 0,021 |
| Occipital_Mid_R | 0,175 | 0,024 | 0,444 | 0,520 |
| Pallidum_L | 0,629 | 0,026 | 0,536 | 0,189 |
| Olfactory_L | 0,743 | 0,027 | 0,669 | 0,397 |
| Olfactory_R | 0,218 | 0,029 | 0,551 | 0,189 |
| Amygdala_R | 0,282 | 0,037 | 0,187 | 0,100 |
| Caudate_L | 0,033 | 0,037 | 0,692 | 0,533 |
| Insula_R | 0,218 | 0,040 | 0,950 | 0,371 |
| Lingual_L | 0,444 | 0,041 | 0,720 | 0,008 |
| Cuneus_R | 0,017 | 0,042 | 0,020 | 0,582 |
| Fusiform_L | 0,073 | 0,047 | 0,003 | 0,003 |
| Temporal_Inf_L | 0,326 | 0,050 | 0,539 | 0,361 |
| Rectus_R | 0,156 | 0,062 | 0,048 | 0,041 |
| OFClat_R | 0,195 | 0,070 | 0,240 | 0,858 |
| Temporal_Pole_Mid_R | 0,651 | 0,072 | 0,768 | 0,632 |
| SupraMarginal_R | 0,391 | 0,073 | 0,416 | 0,638 |
| Temporal_Inf_R | 0,115 | 0,075 | 0,815 | 0,234 |
| Rolandic_Oper_L | 0,374 | 0,076 | 0,138 | 0,109 |
| ParaHippocampal_R | 0,014 | 0,095 | 0,558 | 0,017 |
| Parietal_Inf_R | 0,913 | 0,100 | 0,189 | 0,614 |
| Heschl_L | 0,391 | 0,108 | 0,593 | 0,210 |
| Occipital_Sup_L | 0,815 | 0,118 | 0,532 | 0,224 |
| Pallidum_R | 0,282 | 0,146 | 0,014 | 0,004 |
| Lingual_R | 0,743 | 0,147 | 0,720 | 0,019 |
| Thalamus_medial_R | 0,020 | 0,147 | 0,106 | 0,561 |
| Frontal_Mid_2_L | 0,767 | 0,155 | 0,310 | 0,975 |
| Frontal_Sup_Medial_L | 0,268 | 0,174 | 0,190 | 0,937 |
| OFCmed_R | 0,206 | 0,177 | 0,074 | 0,169 |
| Thalamus_lateral_R | 0,068 | 0,177 | 0,218 | 0,131 |
| Hippocampus_R | 0,165 | 0,205 | 0,329 | 0,792 |
| Occipital_Inf_R | 0,988 | 0,210 | 0,895 | 0,345 |
| Vermis | 0,012 | 0,214 | 0,049 | 0,029 |
| Angular_L | 0,791 | 0,215 | 0,060 | 0,115 |
| Temporal_Pole_Mid_L | 0,101 | 0,223 | 0,168 | 0,129 |
| Temporal_Sup_L | 0,357 | 0,226 | 0,950 | 0,106 |
| Postcentral_R | 0,018 | 0,229 | 0,130 | 0,033 |
| Cingulate_Post_L | 0,815 | 0,231 | 0,005 | 0,167 |
| Precentral_R | 0,326 | 0,236 | 0,357 | 0,253 |
| Fusiform_R | 0,483 | 0,240 | 0,242 | 0,020 |
| Rectus_L | 0,864 | 0,245 | 0,546 | 0,743 |
| Supp_Motor_Area_L | 1,000 | 0,268 | 0,325 | 0,381 |
| Cerebelum_L | 0,185 | 0,297 | 0,633 | 0,312 |
| Temporal_Mid_R | 0,296 | 0,309 | 0,254 | 0,241 |
| ACC_pre_R | 0,743 | 0,329 | 0,272 | 1,000 |
| Temporal_Pole_Sup_R | 0,696 | 0,340 | 0,023 | 0,638 |
| Cerebelum_Crus_R | 0,522 | 0,351 | 0,912 | 0,190 |
| Cerebelum_R | 0,206 | 0,352 | 0,936 | 0,467 |
| Caudate_R | 0,044 | 0,358 | 0,453 | 0,720 |
| Frontal_Inf_Orb_2_R | 0,720 | 0,361 | 0,074 | 0,128 |
| Insula_L | 0,502 | 0,392 | 0,729 | 0,730 |
| Frontal_Sup_2_L | 0,165 | 0,399 | 0,426 | 0,252 |
| Precentral_L | 0,326 | 0,408 | 0,242 | 0,583 |
| Frontal_Med_Orb_R | 0,242 | 0,411 | 0,197 | 0,948 |
| Frontal_Inf_Tri_R | 0,255 | 0,412 | 0,501 | 0,158 |
| Frontal_Sup_2_R | 0,720 | 0,426 | 0,975 | 0,373 |
| Putamen_L | 0,888 | 0,434 | 0,837 | 0,749 |
| Cerebelum_Crus_L | 0,543 | 0,450 | 0,146 | 0,423 |
| Thalamus_medial_L | 0,002 | 0,452 | 0,288 | 0,154 |
| Thalamus_pul_L | 0,357 | 0,452 | 0,719 | 0,708 |
| Cingulate_Mid_L | 0,055 | 0,473 | 0,174 | 0,036 |
| Precuneus_R | 0,095 | 0,473 | 0,055 | 0,052 |
| Frontal_Inf_Orb_2_L | 0,674 | 0,480 | 0,356 | 0,522 |
| Calcarine_L | 0,607 | 0,492 | 0,434 | 0,183 |
| ACC_sub_L | 0,026 | 0,502 | 0,422 | 0,401 |
| Cingulate_Post_R | 0,988 | 0,515 | 0,761 | 0,849 |
| Thalamus_pul_R | 0,195 | 0,529 | 0,696 | 0,337 |
| Frontal_Sup_Medial_R | 0,864 | 0,532 | 0,444 | 0,838 |
| Occipital_Sup_R | 0,185 | 0,543 | 0,206 | 0,573 |
| Heschl_R | 0,988 | 0,558 | 0,859 | 0,795 |
| OFCmed_L | 0,483 | 0,568 | 0,867 | 1,000 |
| Temporal_Pole_Sup_L | 0,963 | 0,580 | 0,281 | 0,662 |
| OFCant_L | 0,502 | 0,583 | 0,375 | 0,275 |
| OFCpost_R | 0,585 | 0,590 | 0,375 | 1,000 |
| Parietal_Inf_L | 0,296 | 0,593 | 0,128 | 0,372 |
| Frontal_Inf_Oper_R | 0,696 | 0,604 | 0,372 | 0,900 |
| Frontal_Med_Orb_L | 0,341 | 0,646 | 0,276 | 0,612 |
| ACC_sup_R | 0,341 | 0,657 | 0,676 | 0,937 |
| Cingulate_Mid_R | 0,963 | 0,696 | 0,938 | 0,521 |
| Paracentral_Lobule_R | 0,463 | 0,707 | 0,765 | 0,754 |
| Paracentral_Lobule_L | 0,651 | 0,708 | 0,876 | 0,950 |
| Frontal_Mid_2_R | 0,720 | 0,708 | 0,218 | 0,670 |
| Temporal_Sup_R | 0,938 | 0,722 | 0,900 | 0,924 |
| Calcarine_R | 0,156 | 0,755 | 0,708 | 0,008 |
| Postcentral_L | 0,206 | 0,755 | 0,399 | 0,876 |
| ACC_sup_L | 1,000 | 0,762 | 0,352 | 0,729 |
| Hippocampus_L | 0,195 | 0,766 | 0,854 | 0,792 |
| SupraMarginal_L | 0,720 | 0,774 | 0,434 | 0,826 |
| ACC_pre_L | 0,767 | 0,787 | 0,754 | 0,862 |
| OFClat_L | 0,938 | 0,789 | 0,502 | 0,458 |
| Parietal_Sup_L | 0,408 | 0,791 | 0,374 | 0,210 |
| Temporal_Mid_L | 0,607 | 0,838 | 1,000 | 0,766 |
| OFCant_R | 0,230 | 0,849 | 0,629 | 0,593 |
| Parietal_Sup_R | 0,696 | 0,851 | 0,766 | 0,790 |
| Rolandic_Oper_R | 0,913 | 0,888 | 0,662 | 0,594 |
| OFCpost_L | 0,651 | 0,891 | 0,496 | 0,766 |
| ACC_sub_R | 0,122 | 0,893 | 0,278 | 0,193 |
| Amygdala_L | 0,138 | 0,919 | 0,674 | 0,525 |
| Frontal_Inf_Oper_L | 0,564 | 0,924 | 0,913 | 0,681 |
| Putamen_R | 0,791 | 0,926 | 0,263 | 0,440 |
| Frontal_Inf_Tri_L | 0,767 | 0,962 | 0,740 | 0,706 |
| Angular_R | 0,206 | 0,962 | 0,142 | 0,177 |
| Supp_Motor_Area_R | 0,743 | 0,963 | 0,543 | 0,356 |
| Precuneus_L | 0,426 | 1,000 | 0,242 | 0,521 |

**Regions related to network disruption and restoration: exploratory analysis**

Since consciousness rather than time was found to account for HDI-Clustering and HDI-Degree changes, we computed the mean HDI-Clustering and the mean HDI-Degree for all the graphs corresponding to the networks of C-group (n=28) and for all the graphs corresponding to the MCS-group (n=11), whatever the time.

After correction for multiple comparison, no region accounted for mean HDI difference between healthy subjects and C-group, healthy subjects and MCS-group and between MCS-group and C-group whatever the metric considered (Clustering, Degree, BC). Consequently, we chose to explore our data at a lower statistical threshold. See below for report of all regional p values considering Clustering, Degree and BC.

We report the graphical representation of the HDI (plots of all regions, Supplementary Fig. 7 for Clustering, Supplementary Fig. 8 for Degree and Supplementary Fig. 9 for BC metrics).

**Supplementary Figure 7:**

Supplementary Fig. 7A represents all the 107 regions mean values giving HDI-Clustering computation corresponding to C-group. Supplementary Fig. 7B represents the same variables for the MCS-group.

These plots evidence some regions with reduced hub properties, that we further named ‘disrupted hubs’. Some nodes presenting no hub behaviour in normal brain networks showed increased metrics’ values in patients’ groups, corresponding to hub behaviour, that we consequently named ‘neo-hubs’. As we observed some regions with medium metric values in healthy subjects’ brain networks which present increased metrics values, we named these ones ‘hyper-hubs’.

**Supplementary Fig. 7A: Plot of healthy subjects’ Clustering regional values vs. difference in Clustering regional values between C-group of sTBI patients and healthy subjects**

The regions are plotted for the C-group (n=28) giving the corresponding HDI Clustering (the slope of the regression line). Circle: regions not accounting significantly for the HDI value, square: regions accounting significantly for the HDI value with p value < 0.01 without correction for multiple comparison. Only statistically significant regions are labelled.

**Supplementary Fig. 7B: Plot of healthy subjects’ Clustering regional values vs. difference in Clustering regional values between MCS-group of sTBI patients and healthy subjects**

The regions are plotted for the MCS-group (n=11) giving the corresponding HDI- Clustering (the slope of the regression line). Circle: regions not accounting significantly for the HDI value, square: regions accounting significantly for the HDI value with p value < 0.01 without correction for multiple comparison. Only statistically significant regions are labelled.

For HDI Clustering, the comparison between MCS and healthy subjects gave the following results for a p < 0.001 without correction for multiple comparison: disrupted hubs were the left and right middle occipital gyri, left inferior occipital gyrus, right cuneus and left and right fusiform gyri; no neo nor hyper hub was identified.

For HDI Clustering, the comparison between C-group and healthy subjects gave the following results for a p < 0.001 without correction for multiple comparison: disrupted hubs were left and right fusiform gyri, no neo nor hyper hub was identified.

For HDI Clustering, the comparison between MCS and C-groups shows no hub disruption but the left parahippocampal gyrus was a neohub in MCS subgroup for a p < 0.001 without correction for multiple comparisons.

**Supplementary Figure 8:**

**Supplementary Fig. 8A: the regions are plotted for the C-group (n=28) giving the corresponding HDI Degree; panel B: the same regions for the MCS-group (n=11).** Circle: regions not accounting significantly for the HDI value, square: regions accounting significantly for the HDI value with p value < 0.01 without correction for multiple comparison. Only significant regions are labelled. Note that the vertical scale is slightly different between panel A and B.

**Supplementary Fig. 8B**

**Supplementary Figure 9:**

**Supplementary Fig. 9A: the regions are plotted for the C-group (n=28) giving the corresponding HDI BC.**

**Supplementary Fig. 9B: the same regions for the MCS-group (n=11).** Circle: regions not accounting significantly for the HDI value, square: regions accounting significantly for the HDI value with p value < 0.01 without correction for multiple comparison. Only significant regions are labelled. Note that the vertical scale is slightly different between panel A and B.

**Regional reorganization related to the HDI**

Our exploratory results suggest reduced segregation and integration functional connectivity in occipito-temporal associative cortices in MCS patients that partially vanishes with consciousness recovery. This is a surprising observation as one would have expected this phenomenon in higher order areas like the left precuneus^5^. Such reversible hypo-connectivity in associative cortices might be a dynamic adaptative plasticity used to reallocate limited neural resources to higher order areas to sustain minimally consciousness emergence in our participants. This deserves further studies including UWS/VS sTBI for HDI comparison.

**Robustness of construction of graphs in case of lesioned brain**

Among the population studied here, two main types of injuries were observed: the diffuse axonal injury (DAI) that does not lead to direct cortical grey matter impact and DAI plus contusions interesting the cortical grey matter (DAI+C). The potential influence of contusions in the graph was addressed in the following way. In case of a cortical lesion in a given region, we checked the time-serie in the corresponding node. If it mainly contained noise at the scale three of wavelet (which is assessed by a low correlation with the other nodes), then it was unconnected to the rest of the graph and the minimal spanning tree artificially generated spurious edges in the graph. The total number of spurious edges per graph was computed and we did not have to exclude graphs as they all reached the threshold fixed for significant connections (5%). Although the analysis of graph topologies was performed in the same way whatever the type of injury, when possible, the two types were observed separately for visual and statistical comparison.

The hemodynamic response function (HRF) is altered after traumatic axonal injuries (n=10 sTBI at Time 1, abnormal signal in the corona radiata) (Supplementary Fig. 10). This result can be interpreted either as no hemodynamic response change in grey matter either as a differential regional change in grey matter between subjects, leading to no change at group level.

**Supplementary Figure 10:**


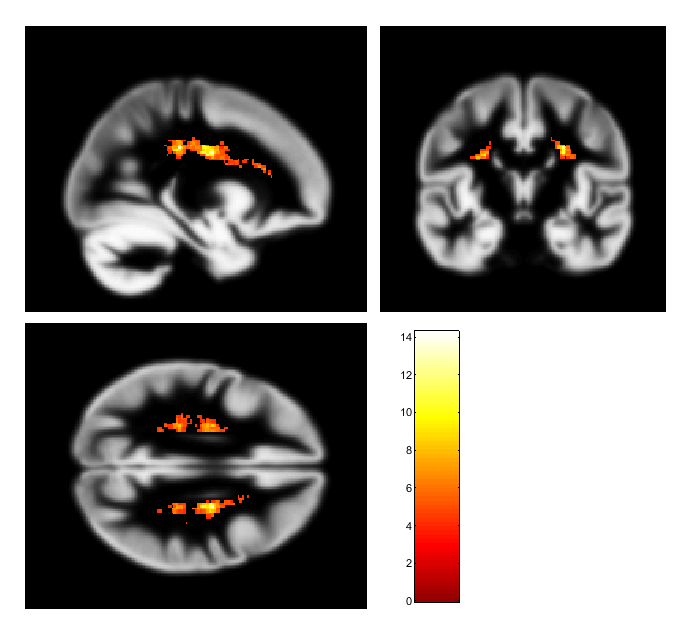


Significant modifications of HRF parameters were observed exclusively in the white matter of trauma patients with respect to controls in the Corona Radiata and in the cerebellar peduncle. HRF amplitude showed a tendency to be increased in the frontal region (C. Jaroszynski, OHBM 2019).

**Supplementary references**

1. Rappaport M, Hall KM, Hopkins K, Belleza T, Cope DN. Disability rating scale for severe head trauma: coma to community. *Arch Phys Med Rehabil*. Mar 1982;63(3):118-23.

2. Achard S, Delon-Martin C, Vertes PE, Renard F, Schenck M, Schneider F, et al. Hubs of brain functional networks are radically reorganized in comatose patients. *Proc Natl Acad Sci U S A*. Dec 11 2012;109(50):20608-13.

3. Power JD, Barnes KA, Snyder AZ, Schlaggar BL, Petersen SE. Spurious but systematic correlations in functional connectivity MRI networks arise from subject motion. *Neuroimage*. Feb 1 2012;59(3):2142-54.

4. Rubinov M, Sporns O. Complex network measures of brain connectivity: uses and interpretations. *Neuroimage*. Sep 2010;52(3):1059-69.

5. Crone JS, Soddu A, Holler Y, Vanhaudenhuyse A, Schurz M, Bergmann J, et al. Altered network properties of the fronto-parietal network and the thalamus in impaired consciousness. *Neuroimage Clin*. 2014;4:240-8.
